# An unexpected large continental source of reactive bromine and chlorine with significant impact on wintertime air quality

**DOI:** 10.1093/nsr/nwaa304

**Published:** 2020-12-28

**Authors:** Xiang Peng, Weihao Wang, Men Xia, Hui Chen, A R Ravishankara, Qinyi Li, Alfonso Saiz-Lopez, Pengfei Liu, Fei Zhang, Chenglong Zhang, Likun Xue, Xinfeng Wang, Christian George, Jinhe Wang, Yujing Mu, Jianmin Chen, Tao Wang

**Affiliations:** Department of Civil and Environmental Engineering, Hong Kong Polytechnic University, Hong Kong 999077, China; Department of Civil and Environmental Engineering, Hong Kong Polytechnic University, Hong Kong 999077, China; Department of Civil and Environmental Engineering, Hong Kong Polytechnic University, Hong Kong 999077, China; Department of Environmental Science and Engineering and Institute of Atmospheric Sciences, Fudan University, Shanghai 200433, China; Departments of Atmospheric Science and Chemistry, Colorado State University, Fort Collins, CO 80523, USA; Department of Atmospheric Chemistry and Climate, Institute of Physical Chemistry Rocasolano, CSIC, Madrid 28006, Spain; Department of Atmospheric Chemistry and Climate, Institute of Physical Chemistry Rocasolano, CSIC, Madrid 28006, Spain; Research Center for Eco-Environmental Sciences, Chinese Academy of Sciences, Beijing 100085, China; Department of Environmental Science and Engineering and Institute of Atmospheric Sciences, Fudan University, Shanghai 200433, China; Research Center for Eco-Environmental Sciences, Chinese Academy of Sciences, Beijing 100085, China; Environment Research Institute, Shandong University, Qingdao 266237, China; Environment Research Institute, Shandong University, Qingdao 266237, China; Univ Lyon, Université Claude Bernard Lyon 1, CNRS, IRCELYON, Villeurbanne 69626, France; School of Municipal and Environmental Engineering, Shandong Jianzhu University, Jinan 250101, China; Research Center for Eco-Environmental Sciences, Chinese Academy of Sciences, Beijing 100085, China; Department of Environmental Science and Engineering and Institute of Atmospheric Sciences, Fudan University, Shanghai 200433, China; Department of Civil and Environmental Engineering, Hong Kong Polytechnic University, Hong Kong 999077, China

**Keywords:** BrCl, reactive halogen, oxidation, coal burning, air pollution, North China

## Abstract

Halogen atoms affect the budget of ozone and the fate of pollutants such as hydrocarbons and mercury. Yet their sources and significances in polluted continental regions are poorly understood. Here we report the observation of unprecedented levels (averaging at 60 parts per trillion) of bromine chloride (BrCl) at a mid-latitude site in North China during winter. Widespread coal burning in rural households and a photo-assisted process were the primary source of BrCl and other bromine gases. BrCl contributed about 55% of both bromine and chlorine atoms. The halogen atoms increased the abundance of ‘conventional’ tropospheric oxidants (OH, HO_2_ and RO_2_) by 26%–73%, and enhanced oxidation of hydrocarbon by nearly a factor of two and the net ozone production by 55%. Our study reveals the significant role of reactive halogen in winter atmospheric chemistry and the deterioration of air quality in continental regions where uncontrolled coal combustion is prevalent.

## Teaser

This study discovered persistent high levels of a reactive halogen compound (bromine chloride, BrCl) which was produced from coal burning and atmospheric photochemistry during winter in northern China, and upon photolysis, BrCl boosts the atmospheric oxidative power and aggravates winter haze and mercury pollution.

## INTRODUCTION

Halogen atoms (chlorine (Cl) and bromine (Br)) can strongly influence the atmospheric chemical composition. High levels of halogens have been shown to deplete ozone (O_3_) in the stratosphere [1] and destroy ground-level ozone of the Arctic [2–4]. There is an emerging recognition that in the troposphere, they can kick start hydrocarbon oxidation that makes ozone, modify the oxidative capacity by influencing the levels of the hydroxyl radical (OH) and hydroperoxyl radical (HO_2_) [5] and perturb mercury recycling by oxidizing elementary mercury (Hg^0^) to a highly toxic form (Hg^II^) [4,6]. Moreover, Cl atoms can remove methane, a climate-forcing agent [7]. While most of the earlier halogen studies focused on the stratosphere and the marine boundary layer, there has been growing interest in the effect of Cl atoms on atmospheric chemistry over continental areas in the last decade because of the existence of anthropogenic chloride sources that can be activated to form Cl atoms [8,9]. Most of the previous studies focused on two Cl precursors, nitryl chloride (ClNO_2_) and molecular chlorine (Cl_2_) [10–13], which were found to enhance ozone formation via Cl oxidation of hydrocarbons [14–16]. However, our knowledge of the abundance and the role of bromine compounds in the polluted continental troposphere is limited. During a recent winter field study in the North China Plain (NCP), we observed surprisingly high levels of bromine chloride (BrCl), which provides a significant source of Br and Cl atoms. We show that intense coal burning and photochemical reactions are responsible for the observed BrCl and other reactive bromine gases. Through model simulations, we reveal that BrCl and other halogens may alter ozone production, hydrocarbon oxidation and conversion of elemental mercury to a soluble form in the surface layer of the atmosphere of the highly polluted NCP.

## RESULTS AND DISCUSSIONS

### Reactive halogen species observations

Our measurements were conducted at the SRE-CAS station [17] in an agricultural field in Hebei Province in the NCP during 9–31 December 2017 (Fig. S1A). The NCP is one of the most populated regions in China and frequently suffers from severe haze pollution during winters [18,19] due to the high densities of human populations and industrial and agricultural activities. Numerous villages in the NCP are within a few kilometers of each other (Fig. S1). The measurement site is surrounded by villages with residents of ∼1000, 1–2 km away from a national highway (G4), 3–4 km away from a provincial road (S335) and ∼10 km southeast of Wangdu township (Fig. S1). During the field measurement, the site was strongly impacted by emissions from road traffic and rural household coal burning for heating and cooking. As a result, extremely high levels of the oxides of nitrogen (NO_x_, 83 ppbv on average) were observed with elevated sulfur dioxide (SO_2_, 14 ppbv on average) and fine particulate matter (PM_2.5_, 137 μg/m^3^ on average). The O_3_ concentrations were low due to removal by high nitric oxide (NO, 53 ppbv on average) (Fig. S2).

**Figure 1.**
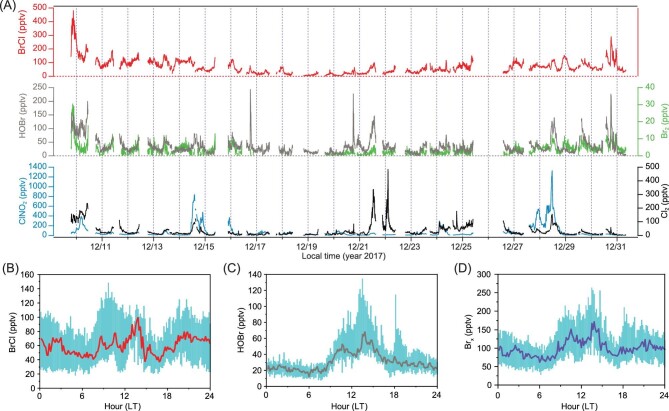
Ambient surface mixing ratios and diurnal profiles of reactive halogen gases at a rural site in NCP during 9–31 December 2017. (A) Time series of Cl_2_, ClNO_2_, HOBr, Br_2_ and BrCl. (B) The diurnal profiles of BrCl for the entire period. The red line is the median, and the cyan shade represents the 25 percentile and 75 percentile values. (C) The diurnal profiles of HOBr for the entire period. The brown line is the median, and the cyan shade represents the 25 percentile and 75 percentile values. (D) The diurnal profiles of gas-phase bromine Br_x_ (= BrCl + HOBr + 2 × Br_2_) for the entire period. The purple line is the median, and the cyan shade represents the 25 percentile and 75 percentile values.

Reactive halogen species (RHS), including BrCl, Cl_2_, ClNO_2_, molecular bromine (Br_2_), and hypobromous acid (HOBr), were measured using a state-of-the-art chemical ionization mass spectrometry (CIMS) technique (see Methods). To our knowledge, this is the first comprehensive measurement of RHS in China. The data reveal three salient features. First, BrCl, a highly photolabile species, frequently exceeded 100 pptv with a maximum value of 482 pptv (10-min average) (Fig. 1A). The maximum value from our study is 10 times larger than the previously reported highest value of 35 pptv in the Arctic [2]. It is also five times higher than the recent aircraft-observed BrCl (up to 80 pptv) in only one out of 50 coal-fired power plant plumes in the northeastern US [20]. Apart from the latter study, BrCl had not been reported in field studies outside of the polar regions [5]. Second, the average HOBr mixing ratio (34 pptv) is also one order of magnitude larger than the level observed in the Arctic [21]. Third, BrCl and HOBr exhibited higher concentrations in the daytime (8 : 00–16 : 00) (local time, LT), while considerable amounts (∼20 pptv) were still present at night (Fig. 1B and C). In terms of other RHS, ClNO_2_ concentrations were lower than the previously observed values in the same area (but at a different location) in the summer of 2014 [14], while the levels of Cl_2_ (Fig. 1A) were comparable to the summer values [16]. Br_2_ was present at very low levels with an average mixing ratio of 4 ppt, which was three times lower than a recently reported value in the Arctic [4] (Fig. 1A). Additional information on the measurement site and ancillary measurements are provided in Supplementary Materials Section 2 and Section 3.

### The source of reactive bromine species

**Figure 2.**
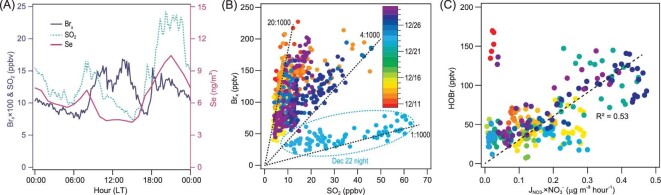
Evidence for the source of the observed reactive bromine gases: coal burning and photo-assisted activation process. (A) Average diurnal profile of Br_x_ (purple line in 10-min average), SO_2_ (green dash line in 10-min average), and Se (red line in 1-hour average) during 9–31 December 2017. The lag in Br_x_ and its daytime peak are due to the photochemical release of Br_x_ from particulate matter. (B) Scatter plot of 10-min average Br_x_ and SO_2_ from 18 : 00 to 09 : 00 when the air masses were stable. Color coded according to sampling date during 9–31 December 2017 (Br_x_ = BrCl + HOBr + 2 × Br_2_). The dotted lines indicate Br_x_/SO_2_ slope (mole/mole) of 1 : 1000, 4 : 1000 and 20 : 1000, covering the range of Br_x_ production from coal burning. Data in the oval depict a case of industrial emissions. (C) Scatter plot of 10-min average HOBr and a proxy of photolysis rate of nitrate (J_NO3−_ × NO_3_^−^ concentration) from 10 : 00 to 15 : 00. J_NO3−_ was calculated from the TUV model (http://cprm.acom.ucar.edu/Models/TUV/Interactive_TUV) under clear sky conditions and then scaled to the measured NO_2_ photolysis rate coefficient (J_NO2_). Color coded according to sampling date during 9–31 December 2017. The outliers in the upper left corner are data from 2 days with the significant impact of fresh emissions or transport from the air aloft (Fig. S7).

**Figure 3.**
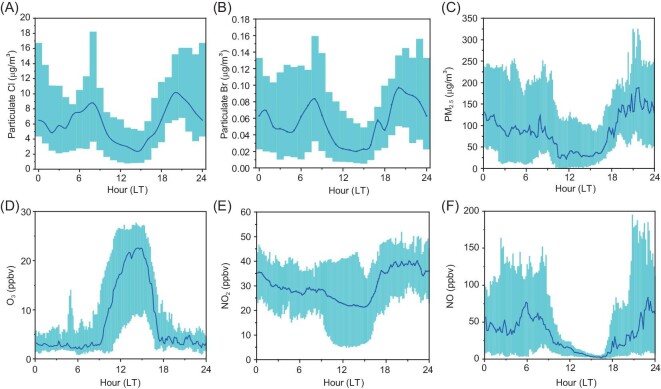
The observed diurnal profiles of trace gases and aerosol at the measurement site in NCP during 9–31 December 2017. (A) Particulate Cl, (B) particulate Br, (C) PM_2.5_, (D) O_3_, (E) NO_2_ and (F) NO. The blue line is the median, and the cyan shade represents the 25 percentile and 75 percentile values.

There is strong evidence that coal burning was a major source of the measured reactive bromine gases, Br_x_ (Br_x _= BrCl + HOBr + 2 × Br_2_). Br_x_ and two coal-burning tracers, SO_2_ and selenium (Se) [22], were elevated in the morning and early evening (Fig. 2A), which is consistent with the increased coal use for heating and cooking during these periods in rural homes according to our on-site survey in the village. Apart from emissions, the concentrations of the measured chemical species would also affect the diurnal changes in the planetary boundary layer height (PBLH), which was not measured during the study period. The PBLH typically is at the maximum in the afternoon and reaches the minimum at night, which means that surface emitted pollutants are expected to undergo more dilution during daytime than at night. Therefore, the morning increase in mixing ratios of Br_x_, the coal burning tracers, and PBLH signify strong emissions from local coal burning, whereas their later larger increase in levels can be partly attributed to decreasing PBLH after sunset. Moreover, the Br_x_ showed a good positive correlation with SO_2_ (Fig. 2B; R^2^ of 0.56 ± 0.17) and Se (Fig. S3A; R^2^ of 0.58 ± 0.26) during the period of intensive coal burning (18:00–09:00) and when air masses were relatively stable (wind speed <3 m/s and no abrupt change in temperature and relative humidity). In addition, particulate halides (chloride and bromide) exhibited the morning and early evening peaks (Fig. 3A and B) and also correlated with SO_2_ and Se (Fig. S3B and C) throughout the campaign. Figure S4A depicts a case of production of HOBr, BrCl, chloride and bromide in a fresh coal-burning plume mixed with traffic emission (containing low O_3_) in the evening of 13 December. These results strongly indicate that coal burning was a substantial source of both reactive bromine gaseous (Br_x_) and particle halides observed at our site.

Figures 2B and S3A indicate large variations in the observed ratios of Br_x_ to SO_2_ or Se, which can be explained by their relative content in coal, combustion conditions and atmospheric processing after emission. The unusual case on 22 December showed relatively low levels of Br_x_ (∼50 ppt) but very high loading of SO_2_ (up to 80 ppb), resulting in the lowest Br_x_/SO_2_ ratio (1 : 1000, mole/mole, see Fig. 2B). The very high concentrations of trace metal elements (Mn and Fe) in this case (Fig. S5) reveal that the air mass might be strongly impacted by emissions from coal burning and ore processing in steel industries. The lowest Br_x_/SO_2_ ratios may indicate a smaller emission of bromine relative to sulfur in iron-smelting processes, incomplete activation of bromine in the air mass, or only partial accounting of reactive bromine gases in the measured Br_x_, and more Br_x_ deposited relative to SO_2_ in more aged air from the steel factories (the nearest one is 80 km from the site). In comparison, the highest Br_x_ mixing ratio was observed during the evening (19 : 00–24 : 00) on 9 December (Figs 1A and S6) and had a much larger Br_x_/SO_2_ ratio (13 : 1000, mole/mole), and this and most of the other cases with moderate to high Br_x_/SO_2_ (4 : 1000–20 : 1000) in Fig. 2B could be characteristic of rural coal burning.

There are no reports on the concurrent Br_x_ and sulfur (S) measurements in domestic coal-burned effluent in China, and concurrently measured Br, Cl and S content in Chinese coal. It is, therefore, difficult to link our observed Br_x_/S ratio to that ratio in coal. Nonetheless, we compared the ambient molar (Br_x _+ Br_particle_)/(SO_2 _+ S_particle_) and (Cl_x _+ Cl_particle_)/(SO_2 _+ S_particle_) ratios with the average and range of Br/S [23,24] and Cl/S [23,25] ratios estimated from their reported contents in Chinese coals (see Supplementary Materials Section 4). We found that the ambient ratios were nearly one magnitude higher than the value of Br/S and Cl/S ratios in Chinese coal, suggesting that halogen compounds are released in a much larger proportion compared to sulfur, or that there are other sulfur species that are released during the smoldering phase of coal burning but are not measured [26]. The Br_x_/SO_2_ ratios observed in our study are one to two orders of magnitude higher than the ratio measured in the northeastern US in the exhausts of coal-fired power plants that are not equipped with wet flue-gas desulfurization [20]. This result indicates that large amounts of reactive bromine species could be released from rural domestic coal burning in the NCP region. Based on the average content of Br and Cl in 137 representative Chinese coal samples [23] and the annual coal assumption in the NCP, we estimate that the amount of Br and Cl in coal can account for our observed atmospheric values (see Supplementary Materials Section 5).

Another potential source of RHS is the open burning of crop residues, but this often occurs in summer in the NCP [14]. We did observe high concentrations of particulate potassium—a biomass burning tracer—on 26 December (Fig. S2), but the concurrent levels of reactive halogen gases were not particularly high. Therefore, we propose that rural homes in the NCP, mostly burning coal as the energy source in winter, are the source of reactive halogen species in our study period. While we present strong evidence for coal burning being a significant source of the observed halogens, it is not clear whether BrCl and HOBr are directly emitted or produced within the coal combustion plumes [20].

Figure 2A shows that the Br_x_ (and BrCl (Fig. 1B)) mixing ratios were highest in the afternoon and exhibited a larger fractional increase than SO_2_. Considering that BrCl is rapidly photolyzed (the noon-time photolytic lifetime of ∼4 minutes) and the PBLH increases during the daytime, the increase in Br_x_ mixing ratio reveals a significant additional source facilitated by sunlight in order to sustain the observed Br_x_ levels during the daytime. The main photochemical chain cycle, which has been proposed to explain the elevated daytime Br_x_ in the Polar regions, involves R1–R8 [27].

(R1) \begin{equation*} {\rm{Br}} + {{\rm{O}}_3} \to {\rm{BrO}} \end{equation*}

(R2) \begin{equation*} {\rm{BrO}} + {\rm{H}}{{\rm{O}}_2} \to {\rm{HOBr}} + {{\rm{O}}_2} \end{equation*}

(R3) \begin{equation*} {\rm{BrO}} + {\rm{N}}{{\rm{O}}_2} + {\rm{M}} \to {\rm{BrON}}{{\rm{O}}_2} + {\rm{M}} \end{equation*}

(R4) \begin{equation*} {\rm{BrON}}{{\rm{O}}_2} + {{\rm{H}}_2}{\rm{O}}\,\,\rightarrow{\!\!\!\!\!\!\!\!\!\!{^{^{\rm{aerosol}}}}}\,\,{\rm{HOBr}} + {\rm{HN}}{{\rm{O}}_3} \end{equation*}

(R5) \begin{equation*} {\rm{HOBr}} + {\rm{HCl}}\,\,\rightarrow{\!\!\!\!\!\!\!\!\!\!{^{^{\rm{aerosol}}}}}\,\,{\rm{BrCl}} + {{\rm{H}}_2}{\rm{O}} \end{equation*}

(R6) \begin{equation*} {\rm{HOBr}} + {\rm{HBr}}\,\,\rightarrow{\!\!\!\!\!\!\!\!\!\!{^{^{\rm{aerosol}}}}}\,\,{\rm{B}}{{\rm{r}}_2} + {{\rm{H}}_2}{\rm{O}} \end{equation*}

(R7) \begin{equation*} {\rm{BrCl}} + {\rm{hv}} \to {\rm{Br}} + {\rm{Cl}} \end{equation*}

(R8) \begin{equation*} {\rm{B}}{{\rm{r}}_2} + {\rm{hv}} \to 2{\rm{Br}} \end{equation*}

The heterogeneous multi-step reaction of HOBr with chloride (R5) and with bromide (R6), which occurs on the surfaces of snow or sea-salt aerosols, is thought to be the primary source for the photolabile BrCl and Br_2_, respectively [2,5,28–31]; additional pathways may also exist [5,31]. In the daytime, Br atoms from photolysis of Br_2_ and BrCl initiate the above chain reaction to liberate more halogens (chlorine via R5 and bromine via R6) from the condensed phase, leading to a rapid increase in reactive bromine gases in the daytime (‘bromine explosion’) [32].

In our study, the presence of elevated daytime BrCl and its good correlation with HOBr loss rate (Fig. S8) suggests that process R5 may play an important role at our site. Box model calculations using up-to-date gas-phase chemistry (see Supplementary Materials Section 6) and a simplified halogen heterogeneous reaction scheme (see Supplementary Materials Section 6.2) showed that R5 could account for a significant fraction of the observed BrCl when the model was constrained by the observed HOBr and other measurements (except BrCl). The observed low Br_2_ concentrations indicate the lower importance of R6. The preponderance of R5 over R6 at our site may be due to particulate chloride (7.3 μg/m^3^ on average) concentration being higher than bromide (0.07 μg/m^3^ on average). Because reaction R5 only activates particulate chloride (not bromide), the presently known bromine activation and propagation reactions (R1–R8) cannot explain the increasing Br_x_ concentrations in the daytime, and there should be an additional bromide activation process that produces HOBr or BrCl at our site. Previous laboratory studies [33–35] observed the production of Br_2_ and BrCl when nitrate and halide in ice or snow are illuminated with ultra-violet light, and it was hypothesized that photolysis of nitrate aerosol generates OH radicals, which subsequently activate bromide to produce Br_2_ and BrCl. During our study, we found a moderate correlation between HOBr and the proxy for aerosol nitrate photolysis rates (the product of calculated J_NO3-_ and the measured PM_2.5_ nitrate concentrations) during 10 : 00–15 : 00 (R^2^ = 0.53) (Fig. 2C), which suggests that the photolysis of nitrate laden in particles may be involved in the activation of the bromide to produce HOBr (and further BrCl). The outliers in Fig. 2C are measurements taken on 29 December, when the sunlight intensity was very low and fresh coal-burning plumes predominated (Fig. S7B), and also on 10 December, when downward transport from the residual layer was suggested by the increase in Br_x_ along with the decrease in other pollutants in the morning (Fig. S7A). Thus, these outliers did not reflect daytime chemistry. Given the considerable scattering in the data, we cannot exclude additional chemical or physical processes that may contribute to Br_x_ production. They include activation of bromide by an organic photosensitizer [36].

### Significant impact on atmospheric chemistry

**Figure 4.**
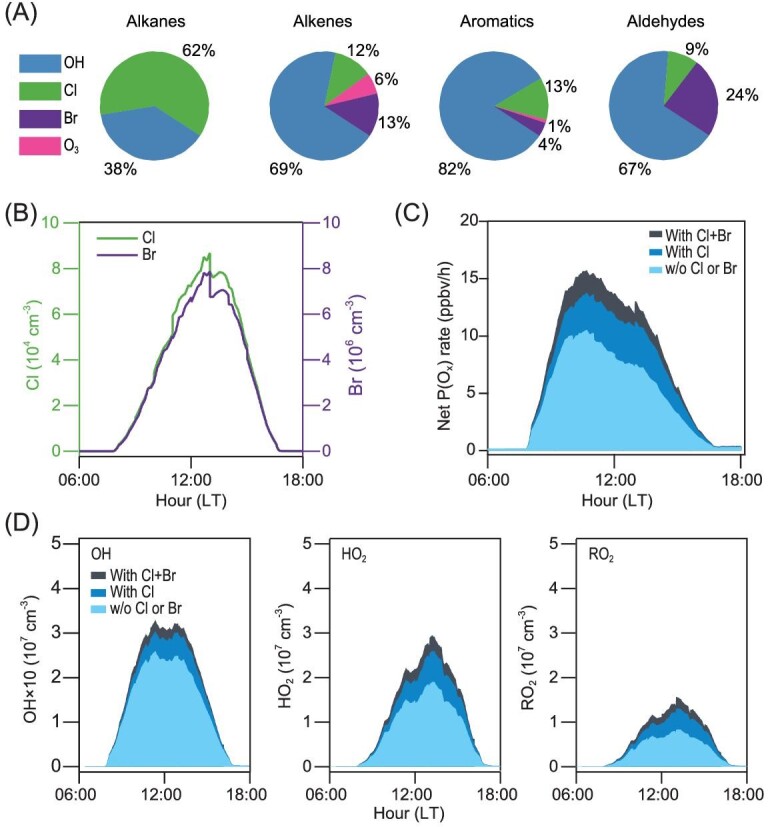
The model calculated contributions of hydrocarbons, ozone production rates and radical abundance averaged for the entire period. (A) Relative contribution to the daily integrated oxidation of alkanes, alkenes, aromatics and aldehydes by OH, Cl, Br and O_3_. (B) The average diurnal profiles of Cl (green line) and Br (purple line) atom concentrations. (C) The average diurnal profiles of the net production rate of O_x_ (different color bars). The light blue bar, blue bar and black bar represent results without Cl and Br chemistry, with only Cl chemistry, and with Cl and Br chemistry, respectively. (D) The average diurnal profiles of OH, HO_2_ and RO_2_ abundances. The light blue, blue and black bars represent the same meaning as panel (C).

Given the high reactivity of Cl and Br atoms, we calculate the impact of the high BrCl and other RHS (Cl_2_, Br_2_, HOBr and ClNO_2_) by using the aforementioned photochemical box model that includes up-to-date Cl and Br gas-phase chemistry by constraining the model with the measured RHS and other relevant observation data (see Supplementary Materials Section 6.1). Because the measured RHS were constrained in the model, the simplified halogen heterogeneous scheme was not used in the calculations of the halogen impact. Photolysis of BrCl was the dominant source of Cl atoms (∼56%), which was 14 times higher than the contribution from ClNO_2_ and two times larger than that from Cl_2_ (Fig. S9A). The model predicted that Cl atoms reached a maximum concentration of about ∼9 × 10^4^ cm^−3^ at noon (Fig. 4B), and the average concentration (1.6 × 10^4^ cm^−3^) is 26 times higher than the previously modeled global mean level of 620 cm^−3^ [37]. The peak Cl production rate at our site (∼8 × 10^6^ cm^−3^s^−1^, Fig. S9A) is more than 10 times that from photolysis of Cl_2_ and ClNO_2_ measured in early winter at a ground site near the City of Manchester (UK) [12] and is several times the primary Cl production rate from ClNO_2_ (predominantly) and Cl_2_ observed during an aircraft campaign in the marine boundary layer off the coast of New York City (US) in late winter [13]. The BrCl was also the dominant source of Br (∼55%) at our site, followed by Br_2_ (∼20%) and bromine oxide (BrO, ∼13%) (Fig. S9B). The maximum Br production rate was 1.0 × 10^7^ cm^−3^s^−1^ (Fig. S9B), two orders of magnitude larger than the maximum Br production rate predicted without anthropogenic Br source in polluted coastal areas in the wintertime [38].

We find that the high levels of Cl and Br atoms have a profound impact on the oxidation of volatile organic compounds (VOCs). On average, ∼60% of daily integrated oxidation of alkanes, ∼10% of alkenes, ∼15% of aromatics and ∼10% of aldehyde was oxidized by Cl atoms during the daytime (Fig. 4A) at the observation site. The Br atoms contributed up to ∼15% of alkenes and ∼25% of aldehydes oxidation but negligibly to the alkanes and aromatics (Fig. 4A) since Br reactions with these chemicals are very slow. The reactions of VOCs with Cl and Br atoms produce alkyl peroxy radical (RO_2_), which are then recycled to form HO_2_ and OH radicals, increasing the average concentration of OH, HO_2_ and RO_2_ oxidant radicals by ∼25%, ∼50% and ∼75%, respectively (Fig. 4D). These results indicate that the abundance (and impact) of HO_x_ radicals (OH, HO_2_ and RO_2_) would be significantly under-predicted if the halogen species found in our study are not considered. A recent field measurement at a rural site north of Beijing in January 2016 [39] has shown more than a factor of 1.5, 4 and 5 with regard to under-predictions of OH, HO_2_ and RO_2_ under high NO_x_ conditions. The halogen (BrCl and Cl_2_) induced chemistry could be part of the reason for the under-estimation of HO_x_.

When both direct (by halogen atoms) and indirect (from HO_x_ produced by the halogen atom reactions) oxidation processes are included, the total VOCs oxidation rate increased by ∼180% for alkanes, ∼50% for C_2_–C_6_ alkenes, ∼40% for aromatics and ∼90% for aldehyde. Moreover, the enhanced HO_2_ and RO_2_ increased O_3_ production through reaction with NO. The Cl and Br atoms enhanced the *in situ* net chemical production rate of O_x_ (= O_3_ + NO_2_) (see Supplementary Materials Section 6.1) by 55% despite destroying ozone at the same time (Fig. 4C). Within these increases, Br atoms enhanced ∼10% for OH, ∼15% for HO_2_, ∼20% for RO_2_ and ∼20% for net O_x_ production rate (Fig. 4C and D). The result indicates that unlike the polar and marine environments where hydrocarbons are low and lead to ozone destruction, the Br atoms in the presence of large hydrocarbons can increase ozone production in polluted continental regions.

The halogen-initiated chemistry can also enhance secondary aerosol formation from oxidations of VOCs. The oxidation of VOCs by radicals leads to secondary organic aerosol (SOA) formation via further reactions of RO_2_ and OH to form low-volatility molecules [40]. Therefore, the halogen atoms, which have been shown to increase the RO_2_ abundance by 75% on average, will significantly increase SOA production. In addition, the halogen-enhanced HO_x_ can increase the production of other secondary aerosol observed during the haze events such as sulfate (by boosting SO_2_ oxidation with enhanced OH, O_3_ and hydrogen peroxide (H_2_O_2_)) and nitrate (via increasing NO_x_ oxidation by OH and O_3_ to form nitric acid) [40]. Therefore, the inclusion of halogen sources discovered in our study in chemistry-transport models is likely to better predict the extent of winter haze formation in North China.

The large abundance of Br atoms can also significantly increase the conversion of airborne elemental mercury (Hg^0^) into reactive mercury (Hg^II^). Hg^II^ is more soluble and hence more prone to deposition to the surface than Hg^0^ and is the main mercury species that deposits and enters ecosystems [41]. Therefore, enhancing atmospheric oxidation would increase Hg^II^ concentrations and deposition to the environment near the source. At our site, the atmospheric lifetime of Hg^0^ due to oxidation by OH or Cl is estimated to be longer than 70 days using the reaction rate coefficients reported by Ariya *et al.* [42]. However, the lifetime is dramatically shortened to only ∼2 days when the average Br atom concentration of 1.5 × 10^6^ cm^−3^ observed during the field study is used. These lifetimes are much shorter than the global mercury lifetime of 10–13 months [41]. Given that coal burning also co-emits a large quantity of mercury [43] and the NCP has one of the highest surface concentrations of Hg^0^ in the world [41], the fast bromine-induced Hg^II^ formation and subsequent deposition may significantly increase the risk for human health and surface ecosystems in the NCP. Future studies are needed to extend our near-field measurements, and modeling of the impact of halogens, to other parts of the boundary layer and downwind regions where coal burning is common.

### Long-term and broad implications

Although the above results are based on observations from one site, we suggest that these findings apply to a large portion of China where coal burning is used to heat homes, especially in rural areas. In 2017, 17 million households in Hebei province used coal as one of their energy sources. The Chinese government projects the four provinces (Hebei, Shandong, Shanxi and Henan) and two municipalities (Beijing and Tianjin) in the NCP to account for 30% of China's total coal consumption in 2020 [44]. Therefore, BrCl is expected to be ubiquitous over large areas of China with heavy coal burning, which is supported by the observation of elevated levels of Br_x_ (up to 194 pptv) in March 2018 at the summit of Mt. Tai, ∼300 km south of the present site (Fig. S10).

Recognizing a large contribution to air pollution by rural coal burning, the Chinese government has embarked on a massive campaign since the winter of 2018 to replace low-quality coal with natural gas and electricity in rural areas of North China [45]. A recent report [46] finds that while some progress has been made, mainly in regions surrounding Beijing, the conversion campaign has been challenging in northwestern (Shaanxi and Shanxi) and northeastern (Heilongjiang) China due to insufficient natural gas supply, inadequate electricity and the high costs of cleaner energy. Even within the NCP, domestic coal burning in the jurisdictions of Beijing, Tianjin and 26 other cities still accounted for ∼40% and ∼35% of the total emissions of SO_2_ and PM_2.5_ in the winter of 2019, with electricity and heat production from coal-fired plants and other industrial coal burning also contributing significantly [47]. The plunge in air quality in the middle of February 2020 in the NCP despite drastic reductions in traffic and some industrial activities amid the Coronavirus epidemic and the Chinese New Year holiday [47] signified the persistence of coal-burning-induced air pollution. Therefore, coal burning will likely be a long-lasting and important source of winter air pollution. Our study demonstrates that intense coal burning not only emits large amounts of primary pollutants such as particulate and sulfur [48,49], but also promotes the formation of secondary pollutants such as ozone, mercury (Hg^II^) and organic aerosols by releasing highly reactive halogen gases. The finding provides new scientific evidence to strengthen the impetus to replace the use of dirty coal.

We note that as domestic coal burning is not limited to China, it is likely that similar production of halogens occurs in other places where uncontrolled domestic coal burning is common. According to the International Energy Agency, coal production in 2017 accounts for 27.1% of the world's total energy supply [50], and the top 20 coal-consuming countries/economies are distributed over all inhabited continents (Table S1). The dominant use of coal in developed countries such as the United States, Japan and Germany is for electricity generation, where stringent pollution control measures are generally utilized. However, a larger proportion of coal use for non-electricity production [50] and/or lower implementation of pollution control in other countries/economies make coal burning an important source of air pollutants not only in China, but possibly also in India, Russia and South Africa etc. Previous source apportionment of ambient PM_2.5_ [51] and bottom-up emission inventories [9] have indicated that coal burning is a major source of atmospheric chloride, and field measurements in China and the US have observed elevated ClNO_2_-associated air masses that were impacted by emissions of coal-fired power plants [10,20,52]. But only one study observed the presence of elevated BrCl in 1 out of 50 plumes from a coal-burning plant in the US [20]. A better understanding of the sources, sinks and impact of reactive halogen species would enable quantification of the findings in the NCP in other continental regions.

**Figure 5.**
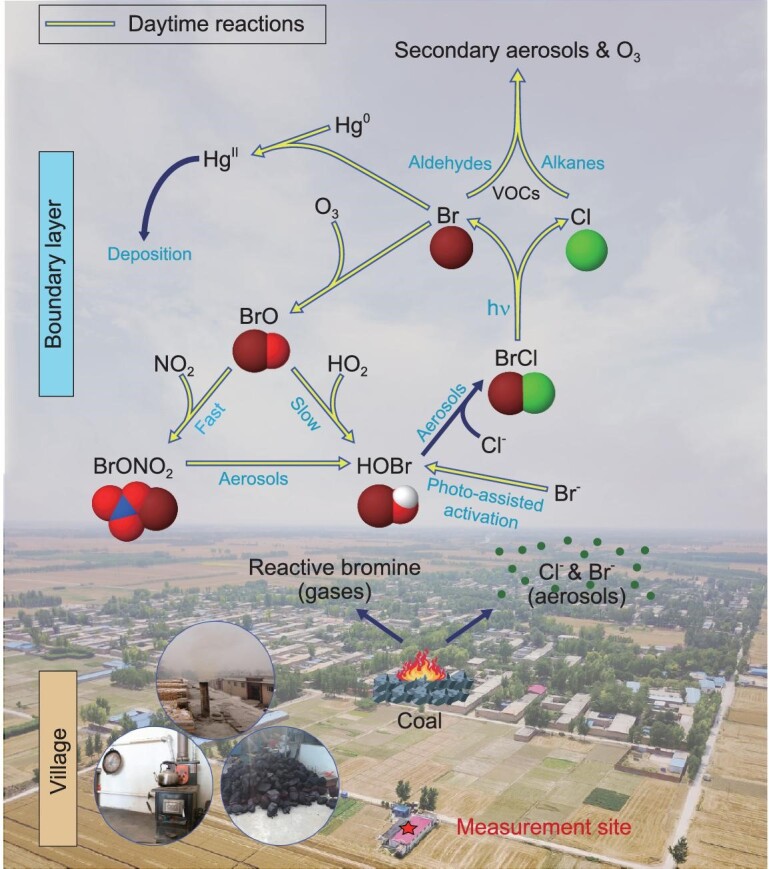
The simplified schematic representation of BrCl source and impact on tropospheric chemistry in North China. Coal burning from rural households emits reactive bromine gases and particulate halogens in both daytime and nighttime. Daytime sunlight-assisted processes, possibly involving nitrate, activate particulate Br to produce HOBr and BrCl. BrCl is also produced by the reaction of HOBr with particulate Cl during the day and night. BrCl is photolyzed to Cl and Br atoms in the daytime. VOCs are oxidized by Cl atoms (mainly on alkanes) and Br atoms (mainly on aldehydes) to produce ozone and secondary aerosols. Moreover, Br atoms significantly accelerate the mercury deposition near the source. All listed halogens are in the gas phase except for Cl^−^ and Br^−^. The three reactions (Br^−^ → HOBr, BrONO_2_ → HOBr, and HOBr → BrCl) are multiphase reactions, which can occur in/on condensed phase. The background photo shows the nearby village and the location of the measurement site (photo credit: Chenglong Zhang and Pengfei Liu).

In summary, we have observed persistent and high concentrations of reactive bromine species (BrCl and HOBr) at ground level in a continental environment. As illustrated in Fig. 5, the large reactive bromine species emerged from coal burning in rural households (and industrial sources) and from daytime chemistry. Photolysis of BrCl significantly increased the levels of Cl and Br atoms. These atoms, in turn, boosted the oxidation rates of VOCs and mercury, and enhanced the abundance of HO_x_ radicals, leading to faster productions of secondary pollutants such as ozone and organic aerosols. They also accelerated the deposition of the toxic form of mercury. Our study reveals that anthropogenic reactive bromine may have larger roles in the chemistry and air quality of the lower troposphere than previously thought, and more research is warranted on reactive halogen species, such as their source(s) and the spatial extent of the role of halogen chemistry, in the polluted continental atmosphere. Our study also suggests the need to control halogens from coal burning, in addition to carbon dioxide, sulfur, nitrogen, particulate matter and mercury.

## METHODS

### Field measurements

Reactive halogen species (including BrCl, HOBr, Br_2_, Cl_2_ and ClNO_2_), dinitrogen pentoxide (N_2_O_5_), other trace gases (including nitrous acid (HONO), H_2_O_2_, SO_2_, carbon monoxide (CO), NO, NO_2_ and O_3_), aerosol concentration and compositions, particle size distributions, VOCs, oxygenated volatile organic compounds (OVOCs), JNO_2_, and other meteorological parameters were simultaneously measured in this study. In this section, we describe in detail the RHS measurements and present information for other measurements in the Supplementary Materials.

A quadrupole chemical ionization mass spectrometer (Q-CIMS) (THS Instruments LLC, Atlanta GA) was used to measure BrCl, HOBr, Br_2_, Cl_2_, ClNO_2_ and N_2_O_5_ by using Iodide (I^−^) reagent ions. The same instrument was used to measure N_2_O_5_ and ClNO_2_ in our previous studies [14,15]. In this study, each target species was monitored at more than two isotopic masses to ensure accurate identifications of ion clusters. BrCl was monitored at 241 amu (I^79^Br^35^Cl^−^), 243 amu (I^79^Br^37^Cl^−^; I^81^Br^35^Cl^−^) and 245 amu (I^81^Br^37^Cl^−^). HOBr was monitored at 223 amu (IHO^79^Br^−^) and 225 amu (IHO^81^Br^−^). Br_2_ was monitored at 287 amu (I^79^Br^81^Br^−^) and 289 amu (I^81^Br^81^Br^−^). Cl_2_ was monitored at 197 amu (I^35^Cl^35^Cl^−^) and 199 amu (I^35^Cl^37^Cl^−^). ClNO_2_ was monitored at 208 amu (I^35^ClNO_2_^−^) and 210 amu (I^37^ClNO_2_^−^). The BrO results are not shown here as we found the BrO measurement suffered mass spectral interference as indicated by the very weak correlation of the observed masses at 222 amu and 224 amu. Hourly scans of the mass spectrum showed that the signal strength for BrNO_2_ (252 amu, 254 amu) was below the CIMS’s detection limit.

The CIMS instrument was housed in a single-story shelter. The sample line was a 3.5 m long PFA-Teflon tubing (1/4 inch outer diameter), with the sampling inlet approximately 1.5 m above the rooftop. We tried to minimize potential inlet artifacts by (i) configuring the sampling inlet system (Fig. S11) to divert large particles from the sample inlet into a by-pass flow and reducing the residence time of the measured gases below 0.5 seconds; and (ii) changing and washing the entire sampling inlet every day to reduce the deposition of Cl^−^ and Br^−^ containing particles on the inlet wall. There were no noticeable changes in the HOBr and BrCl signals when the tubing was replaced (Fig. S4B and C), strongly suggesting the absence of significant heterogeneous reactions in the sample line after using the inlet for a day.

The instrument background signal was measured every day by scrubbing ambient air with alkaline glass wool and charcoal, as many inorganic halogens are efficiently removed by this process, which has also been used by other groups for halogen measurements [3,16,53]. The instrument sensitivity for Cl_2_ and ClNO_2_ was determined on site every two days. A Cl_2_ permeation tube was used as the calibration source, and its permeation rate (378 ng/min, variation <5%) was determined before and after the campaign. The sensitivity of Cl_2_ was stable (2.0 ± 0.16 Hz/pptv) (Fig. S12A) with no significant dependence on RH (Fig. S12B). The uncertainty for the Cl_2_ measurement was about 25%. The calibration method of ClNO_2_ has been reported in our previous studies [14,15]. The sensitivities for other halogen species (Br_2_, HOBr and BrCl) were determined according to their sensitivity ratio relative to Cl_2_, which was determined after the field study. The calibration of Br_2_ was similar to Cl_2_, which was achieved by a permeation tube standard. The HOBr was calibrated using the same method described by Liao *et al.* [3]. HOBr was synthesized from the reaction of liquid Br_2_ with a 0.1 M silver nitrate solution (AgNO_3_), and its concentration was calculated from the Br_2_ formation by passing the HOBr standard through sodium bromide slurry (NaBr). The calibration of BrCl was achieved using the method described by Neuman *et al.* [54], which was also used by Liao *et al.* [3] and Le Breton *et al.* [55]. Briefly, the Br_2_ and Cl_2_ permeation tubes were placed in the same oven at 40°C to produce BrCl via reaction of Cl_2 _+ Br_2_→2BrCl. We have confirmed in the laboratory that all the reduction of Br_2_ and Cl_2_ was converted into BrCl. The concentration of BrCl was calculated from the reduction of Br_2_. The sensitivity of Br_2_, BrCl and HOBr was 1.4 Hz/pptv, 1.6 Hz/pptv and 2.1 Hz/pptv, respectively. The measurement uncertainty for Br_2_, BrCl and HOBr was about 25%, 35% and 39%, respectively.

To make sure there was no significant spectral interference for signals of BrCl, HOBr and Cl_2_, we checked their isotopic signals that showed a strong correlation with slopes being close to the respective theoretical isotopic ratio (Fig. S13). Potential artifacts from the inlet or instrument are of critical concern. We have scrutinized all key steps in our CIMS measurements and made sure that the HOBr and BrCl measurements did not suffer significant artifacts. We examined and ruled out five potential artifacts in the inlet or instrument: (i) inlet artifacts from O_3_ heterogeneous reactions, (ii) potential secondary ion chemistry with IO_3_^−^ in the ion chamber, (iii) secondary ion chemistry with IH_2_O^−^ in the ion chamber, (iv) mass spectral influence from SO_2_, (v) inlet artifacts for BrCl measurement from further HOBr reactions. The detailed results are provided in Supplementary Materials Section 1. In short, we did not find evidence of significant interference or artifacts which would undermine our halogen measurements.

### Chemical box model

A zero-dimensional chemical box model was built based on the latest version of the Master Chemical Mechanism v3.3.1 by using the Kinetic Pre-Processor (KPP) [56] on a MATLAB platform. To better represent the halogen chemistry, we modified the mechanisms to include chlorine- and bromine-related reactions. The detailed kinetics data adopted in the model are listed in Table S2 and described in the Supplementary Materials. In this study, we used the model to calculate the impact of Cl and Br atoms on oxidation chemistry (see Supplementary Materials Section 6.1). The model was constrained to observations of RHS (BrCl, HOBr, Br_2_, Cl_2_, ClNO_2_), N_2_O_5_, HONO, O_3_, H_2_O_2_, NO, NO_2_, SO_2_, CO, temperature, aerosol surface area density, J_NO2_, VOCs and OVOCs. Table S4 shows a summary of the input parameters in the model. Other detailed information on photolysis frequencies, dry deposition, the boundary layer height and the wet deposition is described in the Supplementary Materials. We also combined the above gas-phase model with a simplified halogen heterogeneous reaction scheme to estimate the BrCl production (see Supplementary Materials Section 6.2).

## DATA AVAILABILITY

All data needed to evaluate the conclusions in the paper are present in the paper and/or the Supplementary Materials. CIMS measurement data are available by contacting the corresponding author (T.W.). Other measurement data are available by contacting J.C. (jmchen@fudan.edu.cn) and Y.M. (yjmu@rcees.ac.cn).

## Supplementary Material

nwaa304_Supplemental_File
