# Supplementary material for: An unexpected large continental source of reactive bromine and chlorine with significant impact on wintertime air quality
Source: Natl Sci Rev. 2020 Dec 28;8(7):nwaa304. doi: 10.1093/nsr/nwaa304 (PMC8310770; doi:10.1093/nsr/nwaa304)
Supplement: nwaa304_Supplemental_File [file nwaa304_supplemental_file.docx]

**Supplementary Materials for**

**An unexpected large continental source of reactive bromine and chlorine with significant impact on wintertime air quality**

Xiang Peng^1^†, Weihao Wang^1^†, Men Xia^1^, Hui Chen^2^, A.R. Ravishankara^3^, Qinyi Li^4^, Alfonso Saiz-Lopez^4^, Pengfei Liu^5^, Fei Zhang^2^, Chenglong Zhang^5^, Likun Xue^6^, Xinfeng Wang^6^, Christian George^7^, Jinhe Wang^8^, Yujing Mu^5^, Jianmin Chen^2^, Tao Wang^1^*

*Correspondence to Tao Wang (cetwang@polyu.edu.hk)

† These authors contributed equally to this work

**This file includes:**

Supplementary Text

Section S1. CIMS Measurement

Section S2. Measurement Site

Section S3. Other Measurement Instruments Used in the Work

Section S4. Comparison of Ratios of Halogen to Sulfur in Ambient Air and Coals

Section S5. Accounting for Observed Halogens

Section S6. Chemical Box Model

Supplementary Figures: Figure S1 to S15

Supplementary Tables: Tables S1 to S5

References

**Section S1. CIMS Measurement**

A quadrupole chemical ionization mass spectrometer (Q-CIMS) was used to measure BrCl, HOBr, Br_2_, Cl_2_, ClNO_2_, and N_2_O_5._ Iodide (I^-^) was used as a reagent ion, with the ion chemistry described by Liao et al. [1] and Le Breton et al.[2]. Briefly, the iodide ions selectively react with target gases to form iodide clusters, which are detected by quadrupole spectrometry. The ion-molecule reactions are shown in S1-S6. To minimize the fluctuations in ambient RH that may influence the water cluster of I^-^, 20 sccm of N_2_ blowing through a water bubbler was added into the flow-tube of CIMS. The ratio of I^-^ to IH_2_O^-^ was very stable and didn't show significant diurnal variation throughout the campaign. All analyte signals were normalized to the reagent ion (IH_2_O^-^ at amu 145) to avoid the variation of ambient humidity. To improve the sensitivities for the reactive halogen species, we increased the pressure of the flow tube reactor from 20 torrs to 60 torrs by adding an orifice before the vacuum drag pump.

BrCl + I^-^ 🡪 IBrCl^-^ (amu 243, amu 245) (S1)

HOBr + I^-^ 🡪 IHOBr^-^ (amu 223, amu 225) (S2)

Br_2_ + I^-^ 🡪 IBr_2_^-^ (amu 287, amu 289) (S3)

Cl_2_ + I^-^ 🡪 ICl_2_^-^ (amu 197, amu 199) (S4)

ClNO_2_ + I^-^ 🡪 IClNO_2_^-^ (amu 208, amu 210) (S5)

N_2_O_5_ + I^-^ 🡪 IN_2_O_5_^-^ (amu 235) (S6)

Fig. S11 shows the inlet configuration design in the field study. In our study, we tried to minimize potential inlet artifacts by configuring the sampling inlet system, as shown in Fig. S10 to divert large particles from the sample inlet into a by-pass flow. The total inlet flow rate was 16 LPM, with a by-pass flow of 12 LPM. And the residence time of the measured gases was below 0.5 seconds. During the field measurements, the entire sampling inlet was changed and washed every day to reduce the deposition of Cl^-^ and Br^-^ containing particles on the inlet wall. We prepared three sets of sampling tubes, all of the same length. The replaced tubing went through the following cleaning process: first, we pulled a wad of absorbent cotton stained with alcohol more than three times to clean the inside of the tubing. Second, we rinsed the tubing with deionized water in an ultrasonic bath. Third, we flushed the tube with deionized water. Fourth, we passed zero air through the tube to dry it. There were no noticeable changes in the HOBr and BrCl signals between before and after the tubing replacement (Fig. S4, B and C), strongly suggesting that no significant heterogeneous reactions in the sample line after one-day use.

The calibrations of Cl_2_ and ClNO_2_ were conducted on-site every two days. Cl_2_ was calibrated using the same method as described by Liao et al. [1]. The Cl_2_ standard was generated from a permeation tube heated to 40 ℃ and flushed by 20 sccm of nitrogen gas and then diluted in 6 SLPM humidified zero air. The permeation rate of the Cl_2_ permeation tube was determined before and after the field campaign in the laboratory. The Cl_2_ gas was introduced into KI solution (2 wt.%) for 1 hour. The permeation rate of Cl_2_ was calculated from the I_3_^-^ concentration in the solution, which was determined by ultraviolet-visible spectrophotometry at 351 nm. The permeation rate of Cl_2_ before and after the campaign was stable at around 378 ng/min, with a variation of less than 5%. During the field campaign, the sensitivity of Cl_2_ was stable at 2.0 Hz/pptv with a standard deviation of 0.16, as shown in Figure S12A. The Cl_2_ sensitivity remained constant under different RH in the sample air (Fig. S12B). The measurement uncertainty for Cl_2_ was calculated from the variation of the sensitivity during the campaign and the uncertainty of permeation tube source, and it was about 25%. The calibration method of ClNO_2_ has been reported by several previous studies [3, 4].

The detection sensitivities for other halogen species (Br_2_, HOBr, and BrCl) were determined according to their detection sensitivity ratio relative to Cl_2_ after the field study. The calibration of Br_2_ was achieved with a Br_2_ permeation tube standard. The permeation rate of the Br_2_ permeation tube was also calibrated by passing Br_2_ through the KI solution after the field campaign. The permeation rate of Br_2_ was 730 ng/min under 40 ℃. The HOBr was calibrated using the same method described by Liao et al. [1]. Briefly, HOBr was synthesized from the reaction of liquid Br_2_ with a 0.1 M silver nitrate solution (AgNO_3_). A 20 sccm dry N_2_ was flowed through the solution and then diluted into 6 SLPM humidified zero air. The concentration of HOBr was calculated from the Br_2_ formation by passing the HOBr standard through sodium bromide slurry (NaBr). The conversion rate was above 95%, as determined by the CIMS measurement of HOBr. The calibration of BrCl was achieved using the method described by Neuman et al. [5], which was also used by Liao et al. [1] and Le Breton et al. [2]. Briefly, the Br_2_ and Cl_2_ permeation tubes were placed in the same oven at 40 ℃ to produce BrCl via reaction of Cl_2_+Br_2_🡪2BrCl. The concentration of BrCl was calculated from the reduction of Br_2_. This method assumes that all the decreased Br_2_ is converted into BrCl. To confirm this, we first measured the Cl_2_ and Br_2_ concentrations individually before mixing them. Then we measured the sum concentration of Cl_2_+Br_2_+BrCl in the mixture gas by using the same KI solution absorption method, which was equal to the sum of Cl_2_ and Br_2_ when measured individually. This result confirmed that all reduction of Br_2_ and Cl_2_ were converted into BrCl. The sensitivity of Br_2_, BrCl, and HOBr was 1.4 Hz/pptv, 1.6 Hz/pptv, and 2.1 Hz/pptv, respectively. The measurement uncertainty for the Br_2_, BrCl, and HOBr was about 25%, 35%, and 39%, respectively.

The instrumental background signals were determined every day and subtracted from the total signals to quantify the mixing ratios of BrCl (243 amu), HOBr (223 amu), Br_2_ (287 amu), Cl_2_ (197 amu) and ClNO_2_ (208 amu). The background signal was measured by scrubbing ambient air with alkaline glass wool and charcoal, as many inorganic halogens are efficiently removed by this process, which has also been used by other groups for halogen measurements [1, 6, 7]. The background for HOBr and BrCl was small and roughly constant at around 4 pptv during the field campaign, and did not show dependence on the exact time when the test was conducted during 07:00-21:00 (Fig. S12, C and D). The 3-σ detection limit was 7 pptv for BrCl (243 amu), 6 pptv for HOBr (223 amu), 3 pptv for Br_2_ (287 amu), 3 pptv for Cl_2_ (197 amu) and 5 pptv for ClNO_2_ (208 amu).

To ensure accurate identifications of the ion clusters, we examined the isotopic ratios of the detected halogen species. As the isotopic signals had a strong correlation and the slopes were close to the respective theoretical isotopic ratio, it is confirmed that the detected signal for BrCl, HOBr and Cl_2_ had no significant interference. BrCl was monitored at three masses: 241 amu (I^79^Br^35^Cl^-^), 243 amu (I^79^Br^37^Cl^-^; I^81^Br^35^Cl^-^) and 245 amu (I^81^Br^37^Cl^-^). A strong correlation (R^2^= 0.91) was found between 243 amu and 245 amu (Fig. S13C) with a slope of 0.26, which is close to the theoretical ratio of 0.24. However, a weak correlation between 241 amu and 243 amu and much higher 241 amu to signal 243 amu ratios (compared to their theoretical ratio of 0.77) for some data points indicate some interferences for BrCl measurement at 241 amu (Fig. S13B). Therefore, we used the 243 amu signals to quantify BrCl concentrations. Two masses at 223 amu and 225 amu were used to quantify the mixing ratio of HOBr, and an excellent correlation (R^2^=0.94) was found (Fig. S13D). The slope (0.91) was similar to the theoretical value of 0.98 and that (1.05) reported by Liao et al. (*15*). Cl_2_ was also quantified by two masses at 197 amu (I^35^Cl^35^Cl^-^) and 199 amu (I^35^Cl^37^Cl^-^; I^37^Cl^35^Cl^-^), which showed excellent correlation (R^2^=0.98) with a slope of 0.63 (Fig. S13E) similar to the theoretical value (0.65) and to the ratio (0.65) reported by Liao et al. [1]. An example of a spectrum for CIMS measurement has been shown in Fig. S13A.

To ensure measurement accuracy, it is very important to address potential inlet interferences. We have scrutinized all key steps in our CIMS measurements and made sure that the HOBr and BrCl measurements reflect ambient concentrations and were not the artifacts by examining five potential artifacts from the inlet or instrument: (i) Potential inlet artifacts from O_3_ heterogeneous reactions. Previous laboratory studies [8, 9] showed that O_3_ could react with Br^-^ to produce HOBr. In our field study, the very poor correlation between O_3_ and HOBr, and BrCl suggested that O_3_ was likely not to influence our measurement due to the low levels of O_3_ (the mean value was 4.92 ppbv at night (18:00-09:00), our inlet setup, and daily washing of the inlet tube. We also observed several cases (Fig. S4A) in which high concentrations of HOBr (BrCl) coincided with very low O_3_ in these obvious coal-burning plumes with elevated coal burning tracer (e.g. SO_2_), which provides strong evidence of coal-burning as a source of HOBr and BrCl, but not from O_3_ related reactions. In addition, there were no noticeable changes in the HOBr and BrCl signals between before and after the tubing replacement (Fig. S4B, S4C), strongly suggesting that no significant heterogeneous reactions in the sample line after one-day use. Thus, O_3_ heterogeneous reactions on inlet walls are unlikely as significant artifacts for HOBr and BrCl measurements at our site. A recent measurement study [2] also found a negligible role of ozone for reactive bromine species (HOBr and Br_2_) formation in the sampling inlet. (ii) Potential secondary ion chemistry with IO_3_^-^ in the ion chamber. The hourly mass spectrum scan data showed that 175 amu (IO_3_^-^) was only about 20 Hz during the field measurements, which was much smaller than the signals of primary reagent ions I^-^ (> 50k Hz). At such a low level, the secondary reactions of IO_3_^-^ could not compete with the ion-molecule reactions initiated by I^-^, and thus should have a negligible influence on our measurements. A recent study [10] also found a negligible amount of IO_3_^-^ with ^210^Po as the ion source (similar to our setup), although significant levels of IO_3_^-^ can be formed when electrical discharge was used as the ion source. (iii) Potential secondary ion chemistry with IH_2_O^-^ in the ion chamber. IH_2_O^-^ (at 145 amu) can be easily formed in the I^-^CIMS. We examined the possibility of the formation of IHOBr^-^ from the reaction between IH_2_O^-^ with BrCl or Br_2_ in the ion-molecule reaction chamber. However, we did not observe elevated HOBr signals when we conducted Br_2_ and HOBr calibrations during which the IH_2_O^-^ signals as large as the primary ions I^-^ were present. Thus, this possible reaction does not seem to influence our HOBr measurement. (iv) Potential mass spectral influence from SO_2_. A previous study [11] reported that HOBr suffered interference from ISO_4_^-^ ion at 223 amu in high SO_2_ contained coal-fired power plant plumes. In our CIMS, the two HOBr isotopic peaks (223 amu and 225 amu) were clearly resolved in the field (Fig.S13A), and the ratio of their signal strength was close to the theoretical value (Fig. S13D). Moreover, after our field study, we injected 20 ppb of SO_2_ into the field used tube but did not observe any elevated signals at the mass used to detect HOBr. These results indicated that SO_2_ did not cause interference to HOBr in our CIMS. (v) Potential inlet artifacts for BrCl measurement from further HOBr reactions. Neuman et al. [5] suggested that HOBr could be converted to Br_2_ on their inlet surfaces. A recent airborne measurement has also quantified HOBr loss into instrument and inlet walls using a PFA flow tube inlet system, which varied in length from 0.2 m to 2 m. They found that up to 15% of HOBr was seen to convert to Br_2,_ and this value did not vary with inlet length [2]. In our study, we tried to minimize this potential interference by configuring the inlet system to separate most of the particles from the gas stream, and by washing and changing the Teflon inlet tubing every day. The post-campaign tests confirmed that the BrCl (or Br_2_) did not suffer from significant interference from HOBr in the sampling inlet during our study. Briefly, the lab tests used two types of Teflon tubing with the same length, one used in the campaign and the other a new tubing. Synthesized HOBr mixed with humidified zero air was first introduced to the CIMS without passing through the tubing. Then, the HOBr/air mixture passed through the tubing before entering them CIMS. The decrease between the HOBr signal and the increase of the BrCl signal induced by the tubing was monitored to measure the conversion of HOBr to BrCl in the tubing. Under the RH condition similar to the field campaign and with the same residence time, we found that 15% and 6% of the HOBr were lost, but only 8% and 3% were converted to BrCl for the used tubing and the new tubing, respectively (Fig. S14). And the conversion of HOBr to Br_2_ was 1.2% and 0.2%, in the used and new tubing, respectively (Fig. S14). Thus, the wall loss of HOBr in our inlet setup was insignificant. We also tested the inlet loss of BrCl in these two tubings. The concentration of synthesized BrCl was measured before and after being introduced through the tubing. We found the BrCl loss was 7% and 3% for the used tubing and new tubing, respectively. A similar test was conducted for Cl_2_, and the result showed a loss of less than 2%.

**Section S2. Measurement Site**

The measurements were conducted in the Station of Rural Environment, Chinese Academy of Sciences (SRE-CAS) (38^◦^39'37.36" N, 115^◦^15'16.05" E), which is located near a village of Wangdu county of Hebei Province in the North China Plain (NCP). The measurement site is 170 km southwest of Beijing (population: 21.5 million), 180 km west of Tianjin (population: 15.6 million), 100 km northeast of Shijiazhuang (population: 10.9 million), and is 200 km from the nearest coastline (Fig. S1A). Numerous villages are densely distributed in the Plains within distances of a few kilometers between them (Fig.S1B). The site is situated in an agricultural field surrounded by villages with residents of about 1000 and is 1-2 km away from a national highway G4 and 3-4 km away from a provincial road S335 (Fig. S1B). In addition, several coal-fired power stations [3] are situated within a radius of 100km, and one iron-steel plant (capacity: 2 mt iron and 2.6 mt steel) is about 80 km southwest of our site. This site had been used by several air-quality studies [12-14].

The present measurement study was conducted on 9-31 December 2017. During this period, the site was affected by three anthropogenic sources, which included traffic emissions, dispersed coal combustion, and biomass burning. The traffic emissions were mainly contributed by the national highway G4 and provincial road S335, with a high volume of heavy-duty diesel trucks on the S335 road during nighttime. The dispersed coal combustion was used by the villagers for residual heating and cooking (Fig. S1, C and D).

During the campaign, the measurement site suffered from heavy air pollution as indicated by extremely high concentrations (10min average) of NO_x_ (up to 496 ppbv), SO_2_ (up to 135 ppbv), and PM_2.5_ (up to 463 μg/m^3^). The NO concentration accounts for 64 % of the NO_x_ on average, indicating substantial fresh emissions (Fig. S2). The high concentrations of NO were observed mainly with winds from the north and at nighttime (20:00 to 09:00, Local Time), pointing the heavy-duty trucks on S335 road as the major source of NO_x_. In comparison, SO_2,_ a coal-burning marker, came from all directions. Its concentrations peaked in the morning and at dusk, same as other coal burning tracers, including particulate Cl (Fig. 3A), particulate Br (Fig. 3B), Se (Fig. 2A). These observations revealed a significant impact of residential heating in the surrounding villages on the measurements of this study, which had also been indicated in a previous study at the same site during winter [14]. Although the measurement period was beyond the harvesting season, open field burning of crop residues was observed occasionally, which was reflected by the elevated levels of K^+^ observed on a few days (Fig. S2). Although the O_3_ levels were low due to the titration effect of high NO, secondary pollutants like O_x_ (=O_3_ + NO_2_) and secondary aerosols such as SO_4_^2-^ and NO_3_^-^ were substantial during several pollution episodes, suggesting considerable oxidation taking place. Indeed, high levels of radical precursor/product such as HONO and H_2_O_2_ were observed. Cl^-^ contributed a relatively high proportion of PM_2.5_ mass (Fig. S2). Detailed analysis of these data will be presented in several other manuscripts under preparation.

**Section S3. Other Measurement Instruments Used in the Work**

Besides measurements of the reactive halogen gases, other trace gases, aerosol compositions, particle size distributions, and meteorological parameters were simultaneously measured. CO was measured with an infrared absorption analyzer (Thermo Model 48i). SO_2_ was measured by a pulsed UV fluorescence analyzer (Thermo Model 43i). O_3_ was measured by a UV photometric analyzer (Thermo, Model 49i). NO and NO_2_ were detected with a chemiluminescence instrument (Thermo, Model 42i). H_2_O_2_ was measured by a wet liquid chemistry fluorescence detector (AERO laser model AL 2021). HONO was measured with a long path absorption photometer instrument (QUMA, Model LOPAP-03).

VOCs were measured online by a gas chromatography-mass spectrometry/flame ionization detector (GC-MS/FID, ZF-PKU-VOC1007, Beijing Pengyuchangya) with a time resolution of 1 hour. In total, 56 non-methane hydrocarbons (NMHCs), 13 oxygenated VOCs (OVOCs), and 27 halocarbons were identified and quantified. The detection limits of these VOCs ranged from 0.001 to 0.015 ppbv. An internal method with 4 specific compounds (bromochloromethane, 1,4-diflurobenzene, chlorobenzene, and 4-bromoflurobenzene) was used for the calibration of the GC/MS in every sample, while an external method with 56 NMHCs (Spectra Gases Inc., USA) was applied to the calibration of the GC/FID every week. More detailed information about this instrument was described in the previous studies [12]. Four OVOCs (HCHO, Benzal, Oxylal, Mxylal) that cannot be detected by GC-MS/FID were measured by the off-line DNPH-Cartridge-HPLC method. The sample was collected every two hours and then analyzed in the lab with a High-Performance Liquid Chromatography method.

The aerosol concentration and composition were measured by several instruments. The PM_2.5_ mass concentrations used in the present work were measured by a standard Tapered Element Oscillating Microbalance system (TEOM 1400A, Thermo Scientific). PM_2.5_ compositions were measured a Time of Flight-Aerosol Chemical Speciation Monitor (Tof-ACSM, Aerodyne Inc.), including NH_4_^+^, SO_4_^2-^, NO_3_^-^, Cl^-^ and organic aerosol, with a time resolution of 10 min. Potassium data were from quartz fiber filters (diameter:90 mm) collected every two hours and were later analyzed by ion chromatography (IC, WAYEE IC6200). Cl, Br, and Se elements were analyzed by an elementary analyzer (Xact 625i, CES), which utilizes energy dispersive X-ray fluorescence technique with a time resolution of one hour. Additionally, Organic carbon (OC) and elemental carbon (EC) were measured with an online OC/EC analyzer (Model-4, Sunset Lab. Inc.) with a time resolution of 1 h.

The 2-hour averaged concentrations of chloride, nitrate, sulfate, and ammonium measured by the Tof-ACSM has been plotted against the corresponding WSIs derived from 2-hour filters, which resulted in harmonization factors of 0.85, 1.15, 1.05 and 1.2, respectively. The harmonized and sequentially 1-hour averaged concentrations of chloride and sulfate concentrations fairly matched with these of element measurements of Cl and S by the Xact instrument with regression slopes of 1.05 ± 0.01 and 0.36 ± 0.00, respectively, both of which were in a reasonable range. The lower harmonization factor for chloride was mostly due to the interferences from the organic fragments at m/z 35 at the site with extremely high organic matter. After the harmonization, the regression slope of predicted ammonium (by chloride, nitrate, and sulfate) against the measured ammonium was 1.11 ± 0.01 with an R^2^ = 0.971. This inter-comparison for Cl between WSIs and Xact instruments indicates that most particulate Cl is in the form of water-soluble chloride.

To obtain the surface area density of aerosols, the dry-state particle number size distribution was determined by a Scanning Mobility Particle Sizer (SMPS) (TSI 3082), covering the size ranging from 16.5 to 800 nm. A diffusion dryer was attached to the SMPS. The ambient (wet) particle number size distributions were calculated based on a size-resolved kappa-Köhler function varied with the relative humidity [15]. Aerosol surface area density was finally calculated with the (wet) ambient particle number size distribution assuming spherical particles [3].

Meteorological parameters, including wind direction, wind speed, relative humidity (RH), pressure, and temperature, were measured with a portable weather station (Model WXT520, Vaisala, Finland). J_NO2_ was measured by 4-pi-jNO2-Filter Radiometer (Metcon Company).

**Section S4. Comparison of Ratios of Halogen to Sulfur in Ambient Air and Coals**

We compared the ratio of halogens to sulfur content in the air and that in coals. Reports on concurrent measurements of Br_x_ and S in domestic coal burned effluent in China are not available in the literature, and very few previous studies measured Br and S content in Chinese coal. One study found Br/S molar ratios ranging from 0.01- 8:1000 in 305 samples of coal produced from various regions of China [16, 17]. The nighttime observed Br_x_/SO_2_ in our study (1-20:1000) is near the top end of the large range of the coal Br/S content. Particulate Br and Cl also correlated with SO_2_ and Se (Fig. S4, B and C), indicating that coal burning was a substantial source of both gaseous and particle halides at our site. When including the Br and S in the particle phase, the molar ratio of (Br_x_+Br_particle_) to (SO_2_+S_particle_) observed at the site varied from 2:1000 to 21:1000, which are also near or above the upper value of Br/S in the Chinese coal samples. The observed Br_particle_/(SO_2_+S_particle_) (0.5-4:1000) showed similar enrichment of airborne Br/S compared to the ratio in the coal. We also calculated (Cl_x_+Cl_particle_)/(SO_2_+S_particle_), where Cl_x_ is the sum of ClNO_2_ + BrCl+ 2Cl_2_, whose values (102-975:1000) are higher than the average value (36:1000) estimated from the Cl measured in 43 coal samples in China [16] and the S content reported in a previous study [18]. (Cl_particle_/(SO_2_+S_particle_) was 98-960:1000). These results seem to suggest that halogen compounds are released in much larger proportion compared to sulfur, or there are other sulfur species, which are released during the smoldering phase of coal burning but are not measured [19]. Our analysis did not consider gaseous HBr and HCl as they were not measured during the study, which could make the proportion of airborne Br and Cl even larger. The measured (Br_x_+Br_particle_)/(Cl_x_+Cl_particle_) at night was 0.02, which compared well with the mean value of Br/Cl of 0.01 in the coal [16].

**Section S5. Accounting for Observed Halogens**

We also estimate that the amount of coal burning during the measurement period in the location can indeed produce such high levels of reactive bromine species, as described in the following. Our measurement site is located at the Dongbaituo Village of Gaoling Township in Wangdu county, Baoding City, Hebei Province, and we choose Gaoling Township as the calculation unit.

According to China Dispersed Coal Governance Report 2017 [20], in the year of 2017, about 0.2 billion tons of dispersed coal used by rural villages for household cooking and heating during winter in China, which accounts for 94% of total household coal use. In Hebei Province, the average daily usage of dispersed coal per family was calculated as 20 kg/day/family. The Gaoling Township contains 23 villages, which has a population of 28000 and occupies an area of 40.3 km^2^. Assuming an average family size of 4, the average coal consumed in the Gaolong township is 3400 kg/km^2^/day. Applying the average content of chlorine (405 ppm) and bromine (9 ppm) from 137 representative Chinese coal samples [16], the emission intensity of Cl and Br is 13909 µg/m^2^/day and 348 µg/m^2^/day. Assuming an average Planetary Boundary Layer Height of 500 m, the daily emitted Cl and Br can contribute the respective ambient concentration of at least 27.8 µg/m^3^ and 0.7 µg/m^3^, respectively. These values are sufficient to account for the averaged observed concentration of total Cl (7.69 µg/m^3^, which includes particulate chloride and reactive chlorine gaseous (=ClNO_2_ + 2×Cl_2_ + BrCl) and that of total Br (0.45 µg/m^3^, including particulate Br and reactive bromine gaseous Br_x_). The observed variations could be due to variations in the halogen content of the coal, in addition to the photochemical processes during the daytime.

**Section S6. Chemical Box Model**

An observation-based zero-dimensional chemical box model was built based on the latest version of the Master Chemical Mechanism v3.3.1 by using the Kinetic Pre-Processor (KPP) [21] on a MATLAB platform. To better represent the halogen chemistry, we modified the mechanisms to include up-to-date gas-phase chlorine and bromine chemistry. The detailed kinetics data of gas-phase reactions adopted in the model are listed in Table S2 and described below.

Most kinetic data for inorganic halogen reactions are the recommended values in the latest evaluation of "Chemical Kinetics and Photochemical Data for Use in Atmospheric Studies," which was published by NASA Panel for Data Evaluation [22], and others are from IUPAC (<http://iupac.pole-ether.fr/index.html/>). Most Cl-initiated degradation mechanisms of alkenes, alkynes, aromatics, aldehydes, ketones, alcohols, and some organic acids are based on Xue et al. [23], and the kinetics data of these reactions are updated from the NIST database (<https://webbook.nist.gov/chemistry/>). Besides, 69 new Cl-initiated degradation mechanism and their kinetics for chloro-carbons, esters, ethers, monoterpenes, DMS, and some aldehydes were added based on the experiment data in the NIST database.

For Br reactions with VOCs, the chemical kinetics data were mostly from the NIST database. For some aldehydes and alkenes, the kinetics data are not available in the database. We follow the approach of the MCM protocol, which is based on the known experimental data to give reasonable estimates for the unknown kinetics [24]. There are two approaches to estimate this unknown kinetics in our model. The first one is to assume the unknown kinetics for some species be the same as other species with a similar structure. For example, the reaction rate of ME3BUT1ENE is considered the same as that for ME2BUT1ENE. The other approach is to assume that the ratio of the reaction rate constant with Br to that with OH is constant and estimate the unknown rate constant (k_2-Br_) as k_2-OH_ multiplied by a generic k_1-Br_/k_1-OH_ ratio. For example, the average k_Br_/k_OH_ ratios for C_2_H_5_CHO, C_3_H_7_CHO, and IPRCHO are 0.39. This value is adopted to estimate the kinetics data for C_4_H_9_CHO, BENZAL, GLYOX, and MGLYOX. Another issue for estimation of unknown kinetics data is the branching ratio, as Br atom could either react with alkene via H-abstraction or Br-addition reactions. The unknown branch ratio is estimated based on the known branch ratio of Cl-initiated reaction or OH-initiated reaction [23].

As an observation-based model, the measured values of HONO, O_3_, H_2_O_2_, NO, NO_2_, SO_2_, CO, temperature, aerosol surface area density, and J_NO2_ were averaged or interpolated every minute and constrained into the model. The VOCs and OVOCs were constrained every hour. Concentrations of CH_4_ and H_2_ were kept constant at values of 2000 ppbv [25] and 500 ppbv [26], respectively. Table S3 shows the chlorine and bromine related photochemical reactions. The photolysis frequencies for nitrate, HONO, O_3_, and other species were calculated from the TUV model (http://cprm.acom.ucar.edu/Models/TUV/Interactive_TUV/) under clear sky condition and then scaled to the measured J_NO2_. The dry deposition process in the model was represented by a first-order loss reaction, using the same parameter described in Xue et al. [23]. And the boundary layer height was set as 100 m for nighttime and 500 m for daytime in the model based on previous mixing layer height measurement results conducted in Hebei during wintertime [27]. The wet deposition was ignored as no rain or snow event occurred during the observation period. The model was run from 19:00 of 9 December to 23:50 of 31 December, and the simulation for the first 24 h was repeated three times to stabilize the intermediate species.

We used the model for two purposes in this study. The main purpose was to evaluate the impact of Cl and Br atoms on oxidation chemistry and all measured halogen gases were also constrained in the model (see the following section S6.1). In addition, we added a simplified halogen heterogeneous reaction scheme to estimate the BrCl production and, in this case, the halogen gases (except BrCl) were constrained (see the following section S6.2).

**S6.1, Simulations of the ambient levels of Cl and Br atoms and their impact on atmospheric chemistry**

The box model that includes the above up-to-date gas-phase chemistry was used to calculate the average concentration and sources/sinks of Cl and Br atoms and was constrained by observed values of RHS, including BrCl, HOBr, Br_2_, Cl_2_, and ClNO_2_ as well as other parameters mentioned above. Because the measured RHS were constrained in the model, the simplified halogen heterogenous scheme was not used. Table S4 shows a summary of the input parameters in the model.

We configured three more model scenarios to assess the impact of Cl and Br atoms on oxidation capacity. In the first scenario, all Cl and Br atoms released from the reactions were set to zero, which is the case without influence from Cl or Br atoms. In the second scenario, all Br atoms released from the reactions were set to zero, while Cl atoms remained the same, giving the result of only influence from Cl atoms. The third scenario included both modeled Cl and Br atoms, representing the influences from both Cl and Br atoms.

Furthermore, we used the box model to calculate the net O_x_ (=O_3_ + NO_2_) production rate. The net O_x_ production rate was calculated by considering both the production and consumption of O_x_. The production rate, p(O_x_), can be calculated from Eq. (1) as the sum of reactions between peroxy radicals and NO, the thermally decomposing or reaction with OH of peroxy nitrate, and the photolysis of ClNO_2_, ClONO_2,_ and BrONO_2_. The consumption rate, l(O_x_), can be calculated from Eq. (2), based on reaction rates for ozone photolysis, reactions of O_3_ with halogen atoms, HO_x_ and alkenes, the reaction of OH with NO_2_. The net O_x_ production rate, P(O_x_), is the difference between the production and loss rate, as shown in Eq. (3).

$p \left( O_{x} \right)= k_{HO_{2}+NO}\left[ HO_{2} \right]\left[ NO \right]+ \sum_{i} k_{RO_{2,i}+NO}\left[ RO_{2,i} \right]\left[ NO \right]+ \sum_{j} k_{RO_{2}NO_{2,j}}\left[ RO_{2}NO_{2,j} \right]+\sum_{j} k_{RO_{2}NO_{2,j}+OH}\left[ RO_{2}NO_{2,j} \right][OH]+ j_{ClNO_{2}}\left[ ClNO_{2} \right]+ j_{ClONO_{2}}\left[ ClONO_{2} \right]+ j_{BrONO_{2}}\left[ BrONO_{2} \right]$ (Eq .1)

$l \left( O_{x} \right)= k_{O(^{1}D)+H_{2}O}\left[ O(^{1}D) \right]\left[ H_{2}O \right]+ k_{OH+O_{3}}\left[ OH \right]\left[ O_{3} \right]+k_{{HO}_{2}+O_{3}}\left[ {HO}_{2} \right]\left[ O_{3} \right]+ \sum_{i} k_{O_{3}+{Alkenes}_{i}}\left[ O_{3} \right]\left[ {Alkenes}_{i} \right]+ k_{OH+{NO}_{2}}\left[ OH \right]\left[ {NO}_{2} \right]+ \sum_{j} k_{RO_{2,j}}\left[ RO_{2,j} \right]\left[ {NO}_{2} \right]+ k_{ClO+NO_{2}}\left[ ClO][NO_{2} \right]+k_{BrO+NO_{2}}\left[ BrO][NO_{2} \right]+ k_{Cl+O_{3}}\left[ Cl][O_{3} \right]+ k_{Br+O_{3}}\left[ Br][O_{3} \right]$ (Eq. 2)

$P \left( O_{x} \right)= p \left( O_{x} \right)-l \left( O_{x} \right)$ (Eq. 3)

**S6.2. Simulation of BrCl mixing ratios**

To assess whether the observed daytime BrCl could be produced by the heterogeneous process of HOBr, we combined the same box model described above with a simplified halogen heterogeneous reaction scheme which treated four heterogeneous reactions as the first-order reaction with a constant value of uptake coefficient (Table. S5). The combined model simulated the BrCl concentration with constrained RHS (including HOBr, Br_2_, Cl_2_, and ClNO_2_) except for BrCl as well as other input parameters described in Table S4. In this stimulation, different uptake coefficients of HOBr were tested, from 0.05 to 0.2. As the box model lacks sinks for BrCl during nighttime, we compared the simulation results with average daytime observation from 11:00 to 14:00. Model calculation indicated that the BrCl daytime concentration could be reproduced (>99%) with the HOBr heterogeneous uptake coefficient of 0.1 (Fig. S15); while other possible pathways, including the gas phase production reactions and the heterogeneous reaction of HOCl/Br^-^ and ClONO_2_/Br^-^, had a negligible contribution to BrCl because their reaction rates 2 to 6 order of magnitude slower than that for HOBr/Cl^-^ and a much lower concentration of bromine than that of chloride at our site. The HOBr uptake coefficient of 0.1 used our model simulation is the same as the laboratory derived HOBr uptake coefficient on sulfuric acid solutions with pH=2 and T=210K [28], and is also comparable with 0.16 calculated using the method proposed by Ammann et al. [29] under the ambient condition at our site ([Cl^-^]=2 M and aerosol pH=5 which is estimated from a thermodynamic model (E-AIM, http://www.aim.env.uea.ac.uk)). It should be noted that our simplified heterogeneous scheme only considered four main heterogeneous reactions and assumed constant uptake coefficients, to better simulate the complex halogen chemistry, future study should employ a multiphase model which considers more comprehensive reactions in/on condensed phase and rigorous treatment of the uptake processes.

**Supplementary Figures**


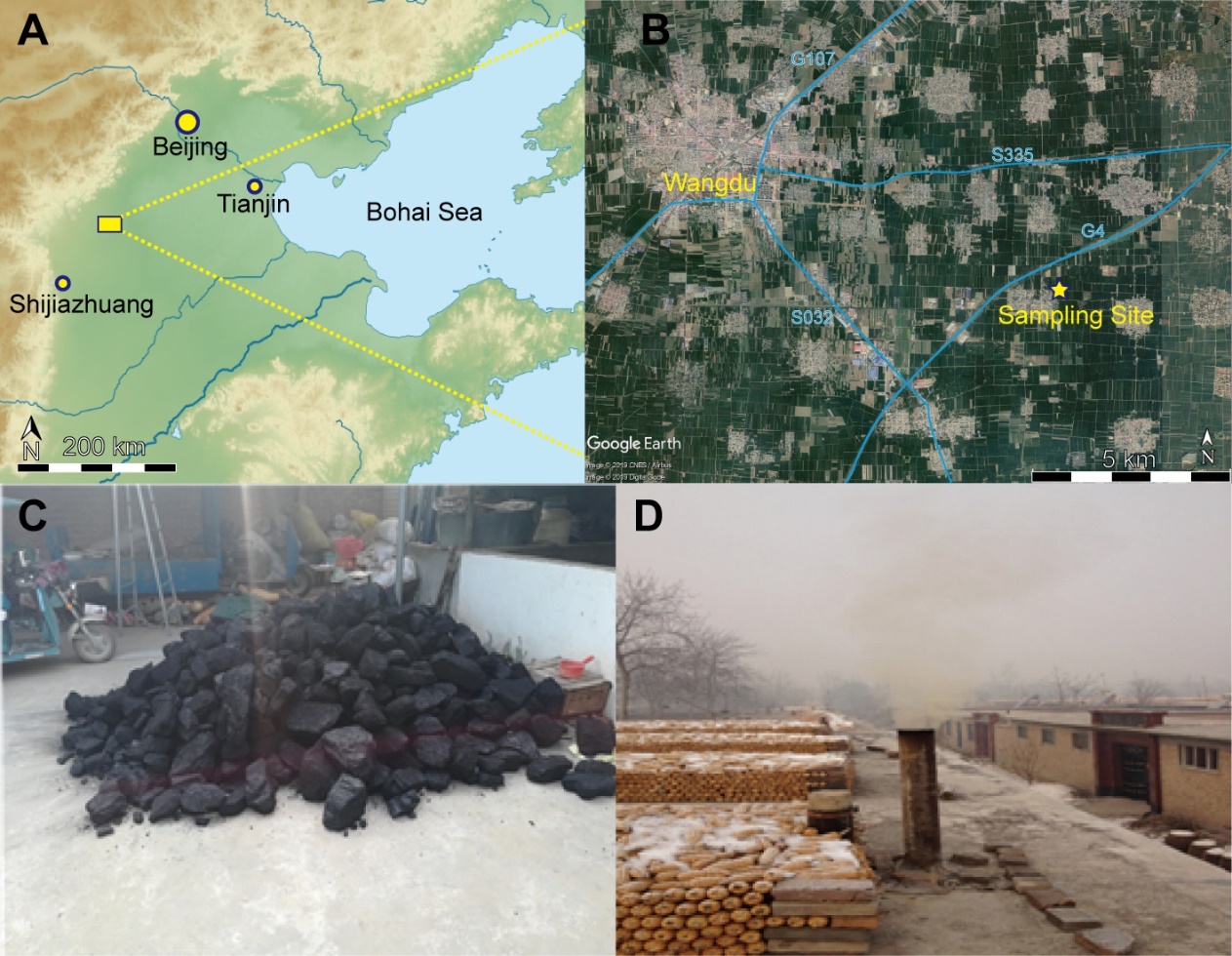


**Supplementary Figure 1: Maps of the study area and the coal burning activity in the nearby villages. (A)** The location of three megacities (yellow cycle) in the North China Plain and the Wangdu township (yellow box). (**B**) The location of the sampling site (yellow star icon) and the surrounding villages and roads. (**C**) The stacks of coal in the courtyard. (**D**) Coal-burning exhausts from the villager’s chimney. (Photo Credit for B: Google Earth; Photo Credit for C and D: Chenglong Zhang and Pengfei Liu; RCEES, CAS).


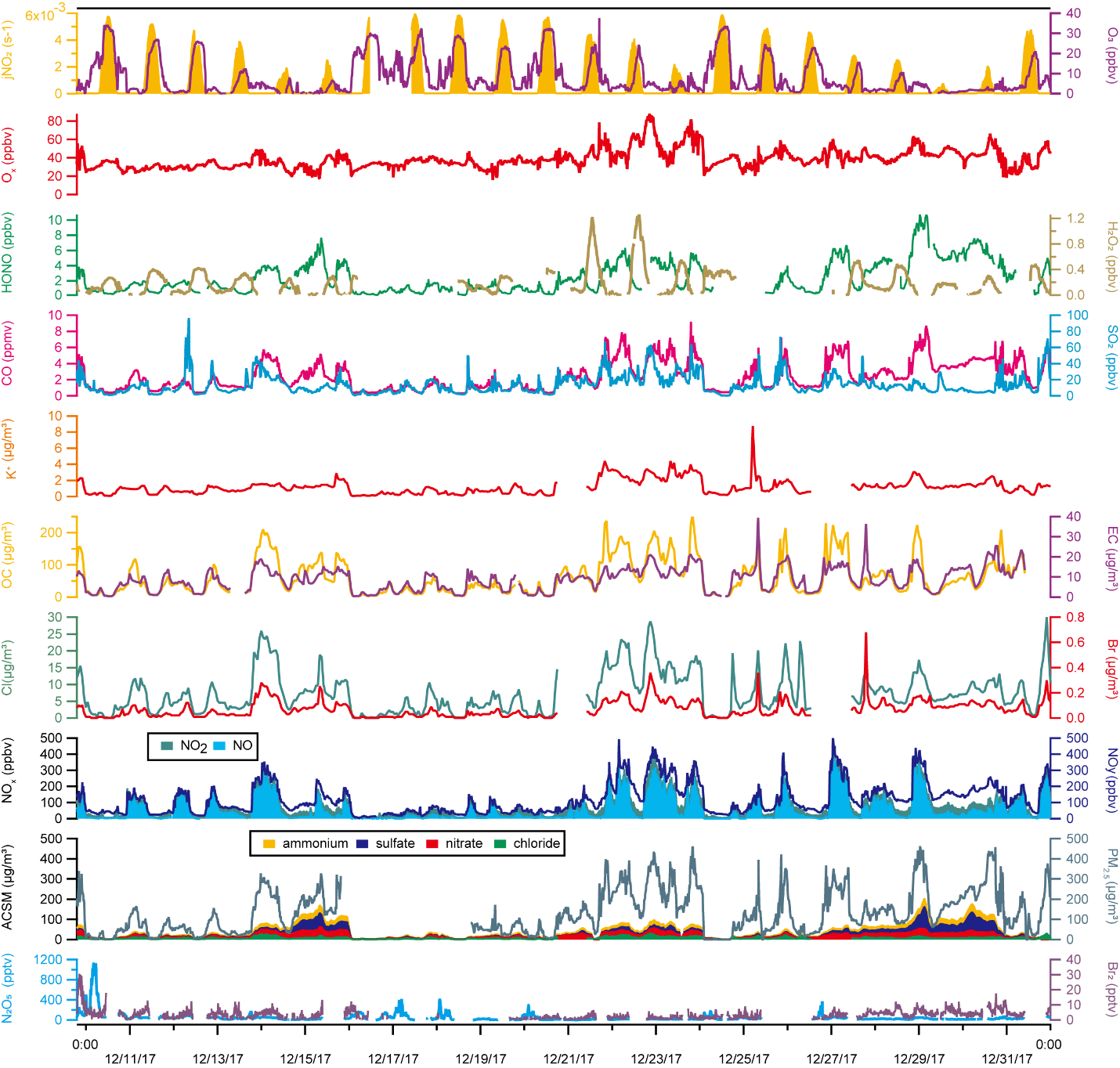


**Supplementary Figure 2: Ambient surface mixing ratios of trace gases and aerosol measured during 9-31 December 2017 at the measurement site in NCP.**


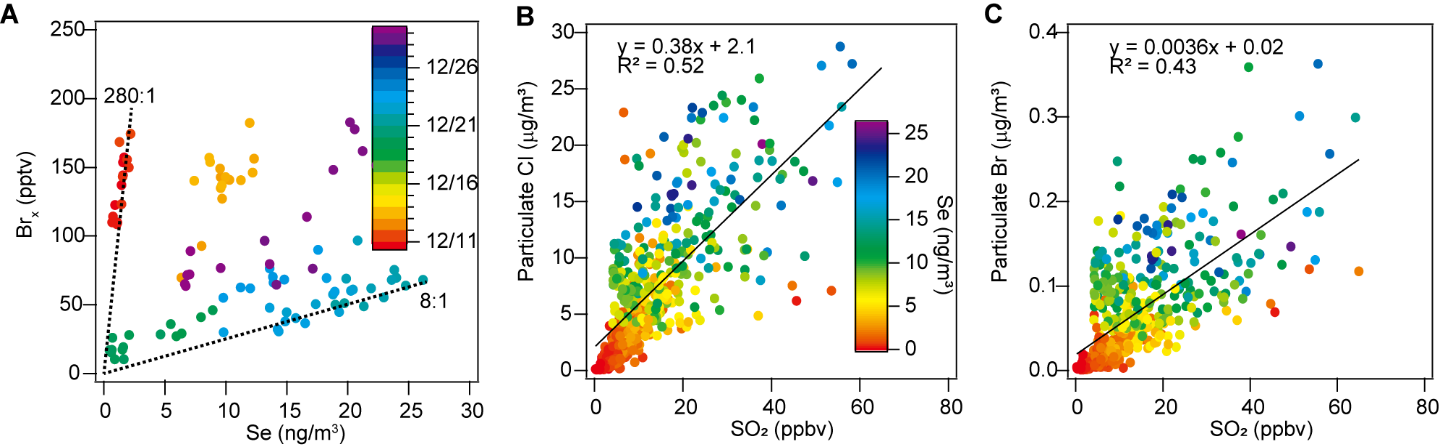


**Supplementary Figure 3: Evidence of coal burning as the source of the observed reactive bromine gases and particulate halide. (A)** Scatter plot of 1-hour average Br_x_ and coal burning markers (Se) from 18:00 to 09:00. The color coded is the sampling date from 9 to 31 December 2017. The average correlation coefficient is 0.58±0.26. (Br_x_ = BrCl + HOBr + 2×Br_2_). The dotted lines indicate Br_x_/Se slope (mole/mole) of 8 and 280. This figure also shows the variability in gas phase Br_x_ due either to variations in the halogen content of the coal and/or the daytime processing. **(B)** Scatter plot of 1-hour average coal burning markers (SO_2_) and particulate Cl for the entire sampling period (the color coded according to the 1-hour concentration of Se). **(C)** Scatter plot of 1-hour average coal burning markers (SO_2_) and particulate Br for the entire sampling period. (the color coded according to the 1-hour concentration of Se)


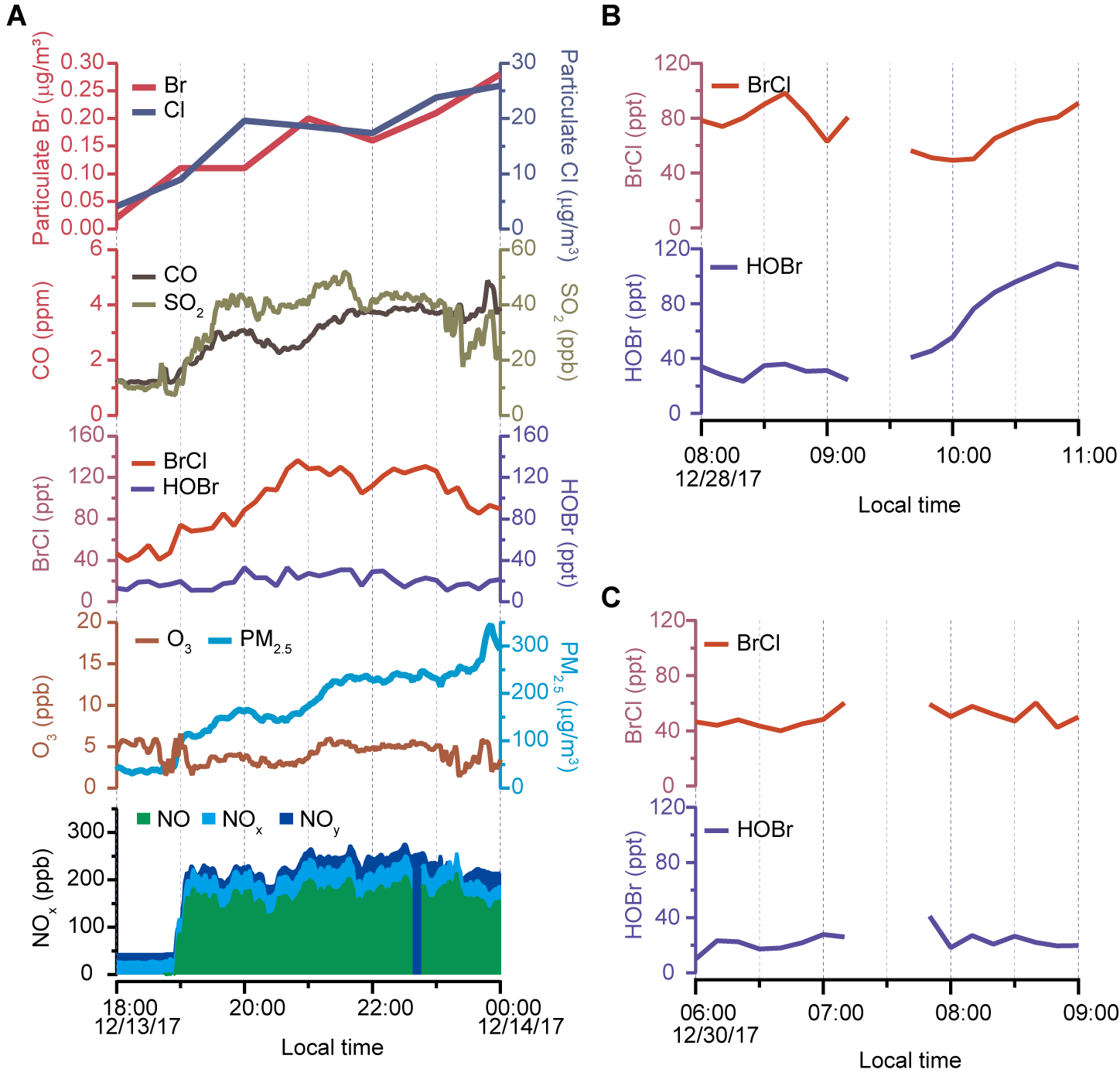


**Supplementary Figure 4: No indication of inlet artifact for HOBr and BrCl measurement.** (**A**) One coal burning case with high ambient BrCl and low HOBr levels but low O_3_. (**B**) and (**C**) is the ambient HOBr and BrCl measurements before and after tube replacement on two days.


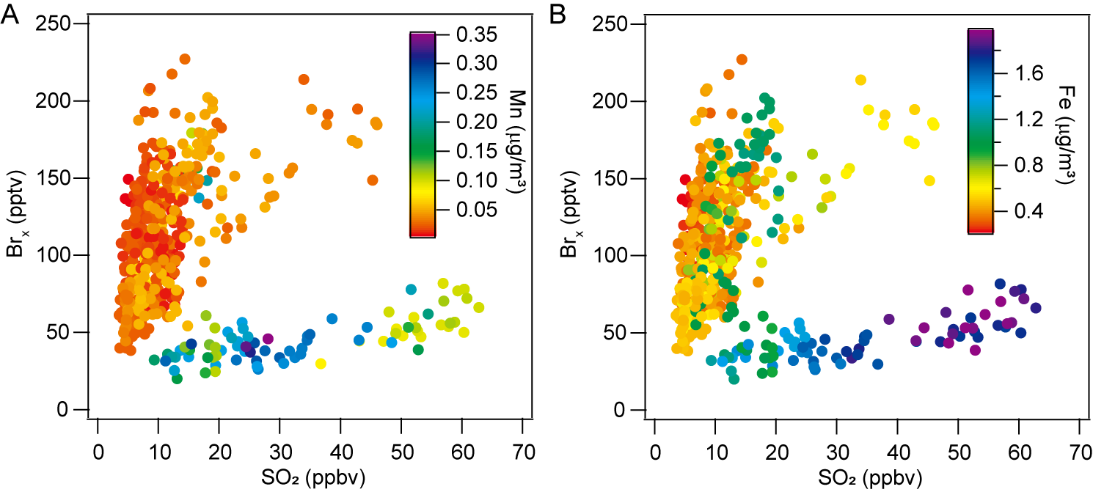


**Supplementary Figure 5: Scatter plot of 10-min average Br_x_ and SO_2_ from 18:00 to 09:00 when the air masses are stable** (Br_x_ = BrCl + HOBr + 2×Br_2_)**.** Color coded according to the concentration of (**A**) elemental Mn, and (**B**) elemental Fe. The data in the oval on the night of 22 December are associated with very high concentrations of Fe and Mn, indicative of the impact of emissions of steel industries in that case. The steel-making process is known to release large amounts of gaseous SO_2_ and particulate enriched in Fe and Mn in the blast-furnace units, which melt iron ores with burning coke [30].


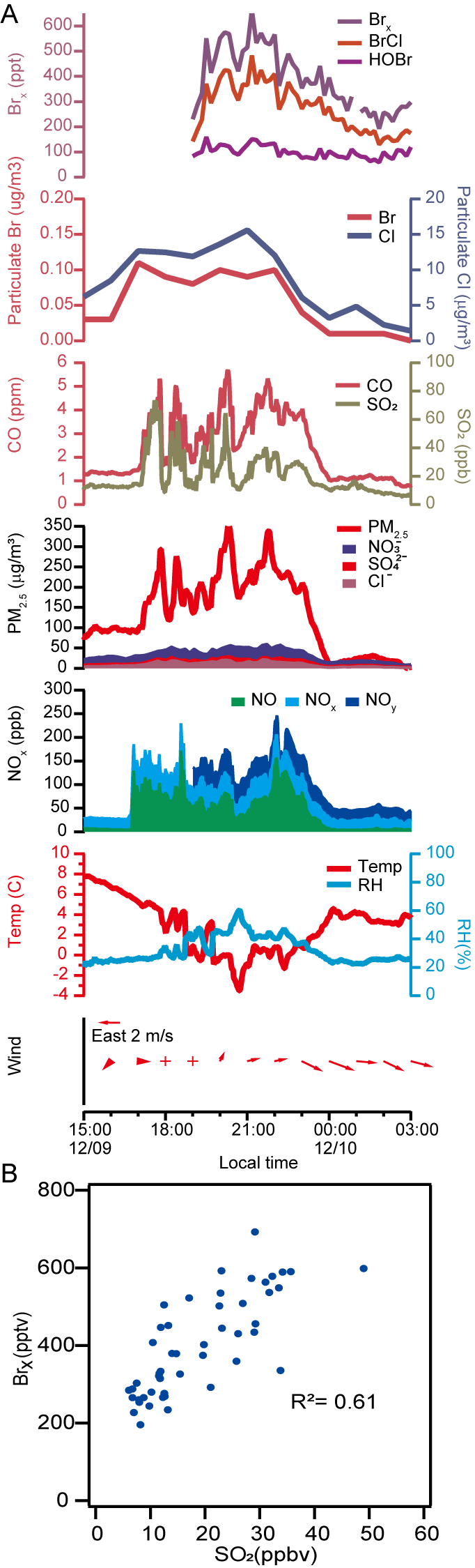


**Supplementary Figure 6: The highest concentration of BrCl on 9 December 2017. (A)** Ambient surface mixing ratios of Br_x_ (=BrCl + HOBr + 2×Br_2_), BrCl, HOBr, SO_2_, CO, other trace gases, aerosol and meteorological data observed from 15:00 on 9 December to 03:00 on 10 December 2017 at the measurement site. **(B)** Scatter plot of 10-min average Br_x_ and SO_2_ from 19:00 09 December to 03:00 10 December.


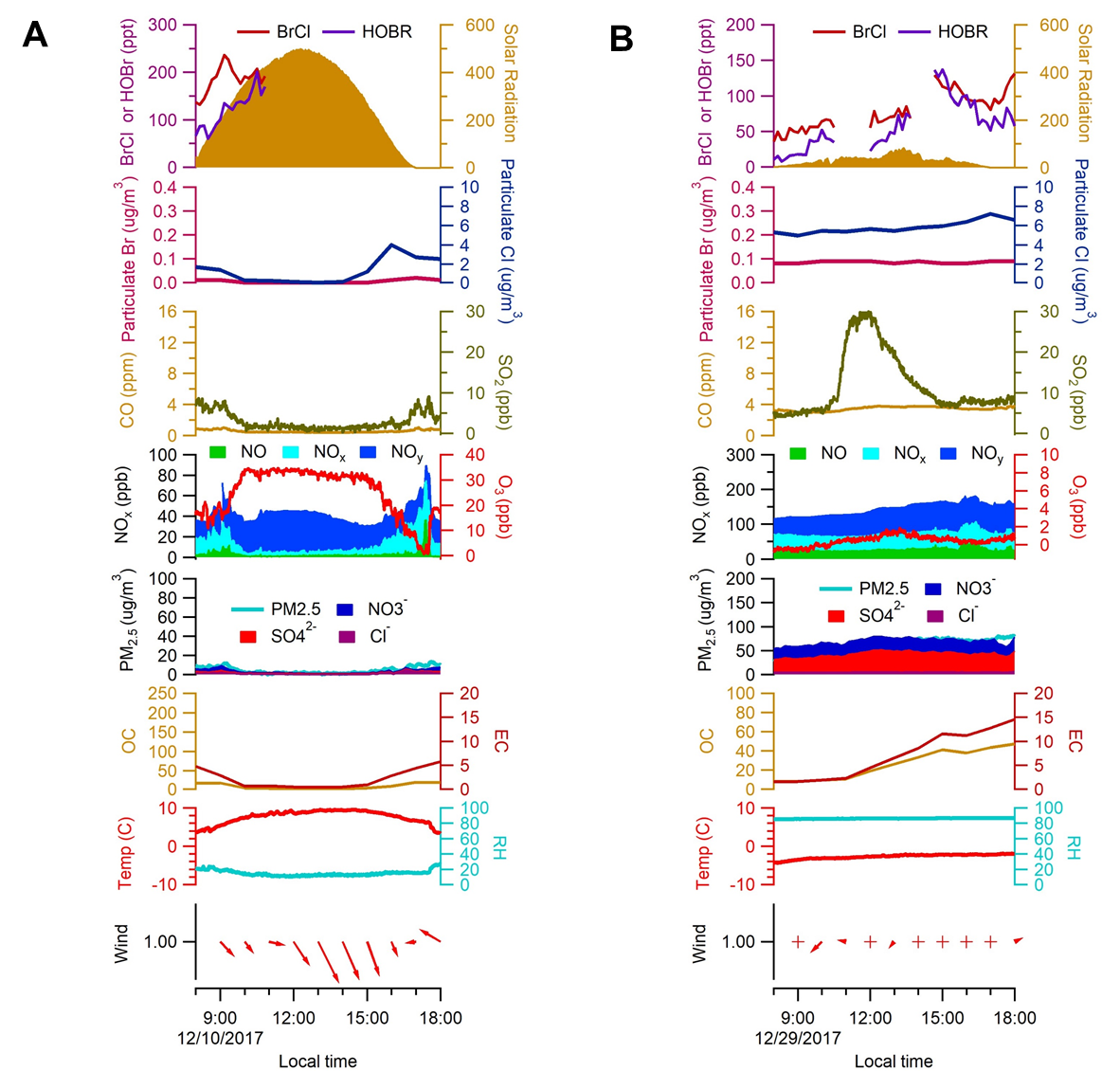


**Supplementary Figure 7: Ambient observations of reactive halogen gases and other parameters on** (**A**) 10 December 2017, and (**B**) 29 December 2017.


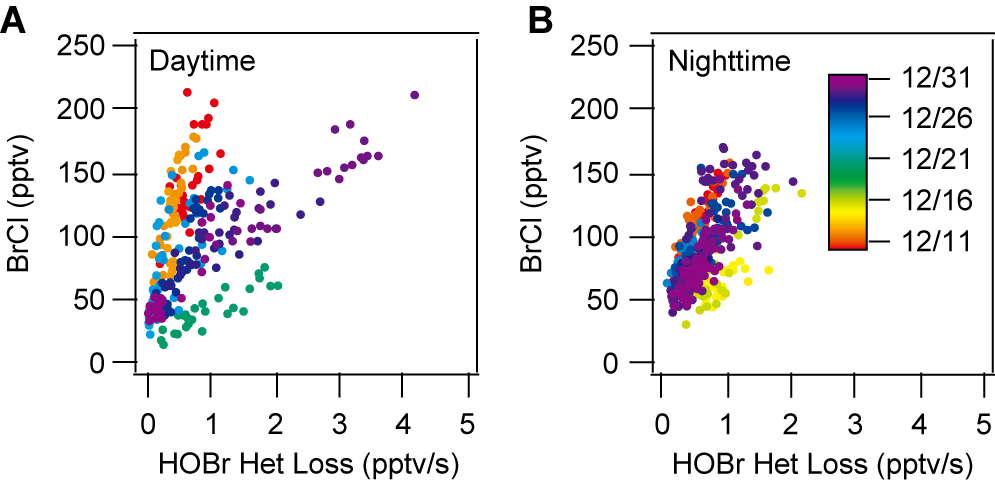


**Supplementary Figure 8: The scatter plot of BrCl and the heterogeneous loss rate of HOBr (**$\boldsymbol{=}\frac{\boldsymbol{1}}{\boldsymbol{4}}\boldsymbol{c}_{\boldsymbol{HOBr}}\boldsymbol{\gamma}\boldsymbol{S}_{\boldsymbol{a}}\boldsymbol{[HOBr]}$**, assuming uptake coefficient** $\boldsymbol{\gamma}$ **= 0.1), Color coded according to sampling date on 9-31 December 2017.** (**A**) during the 10 daytime cases, and (**B**) during the 12 nighttime cases. These cases were selected based on the following criteria: BrCl above 20 pptv, wind speed below 5 m/s, and the case duration longer than 3 hours. The slope of the scatter plot of BrCl concentration and HOBr loss rate varied among the cases, indicating varying (not constant) γ on different days. The value 0.1 was taken a previous laboratory study of HOBr uptake on sulfuric acid solutions with pH=2 and T=210K [28].


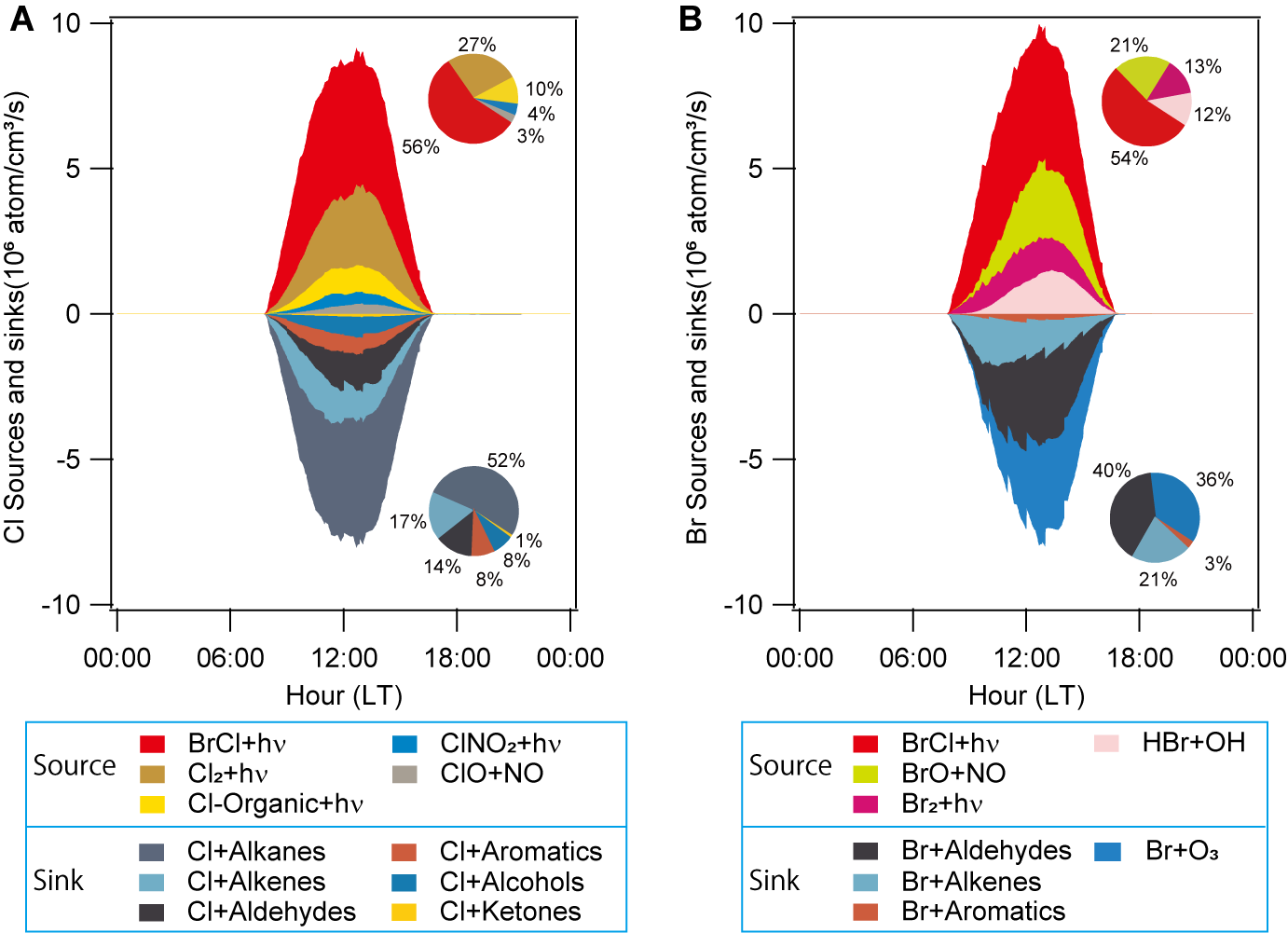


**Supplementary Figure 9: Model simulated major chlorine and bromine production and loss pathways averaged for the entire sampling period. Other pathways with a contribution of less than 0.5% were not shown in this figure.** (**A**) The average diurnal profiles of sources and sinks of the Cl atom. Right upper inset: the daytime average contribution from different sources to Cl atom. Right bottom inset: the average daytime contribution from different sinks to Cl atom. (**B**) The average diurnal profiles of sources and sinks of Br atom. Right upper inset: the daytime average contribution from different sources to Br atom. Right bottom inset: the average daytime contribution from different sinks to Br atom.


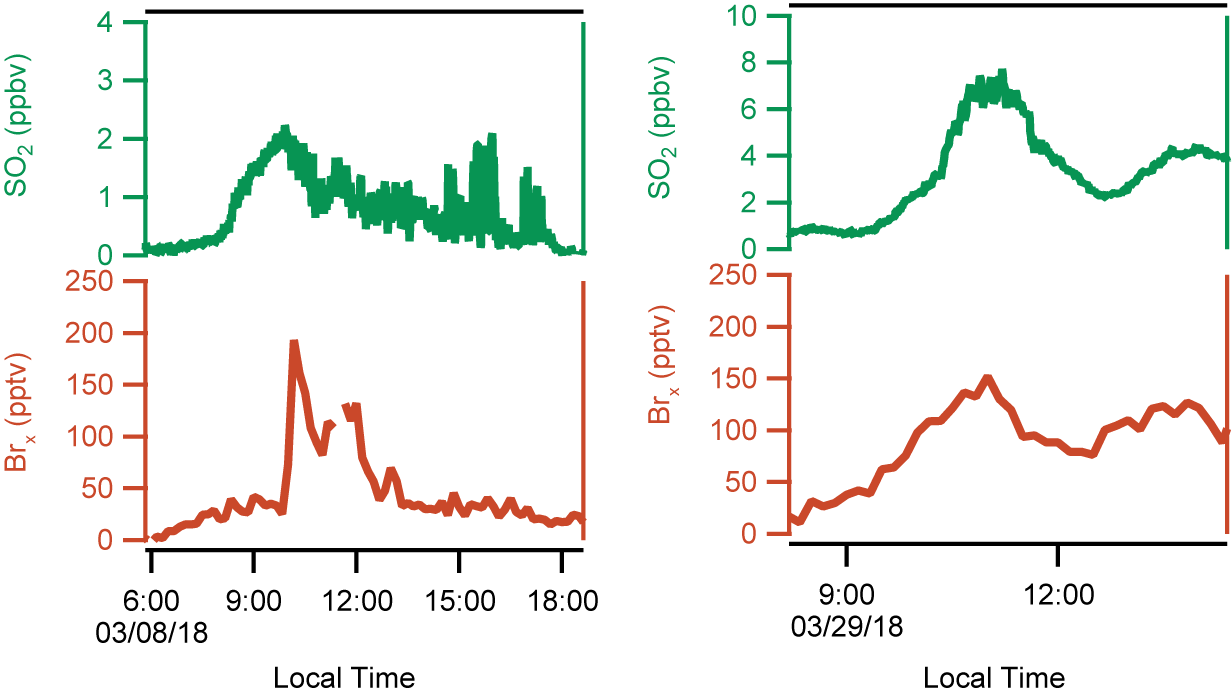


**Supplementary Figure 10: Ambient surface mixing ratios of Br_x_ (=BrCl + HOBr +** **2×Br_2_) and SO_2_ observed on 8 March and 29 March 2018 at a high-altitude site (Mt. Tai, 1465 m a.s.l.) in the North China Plain (NCP).** BrCl, HOBr, and Br_2_ were measured by using CIMS in Mt. Tai. The CIMS configurations and calibration methods used in Mt. Tai were the same as in Wang Du. Mt Tai is 300 km south of Wangdu in Shandong Province. A previous study reported a number of power plant plumes containing elevated concentrations of ClNO_2_ [31].


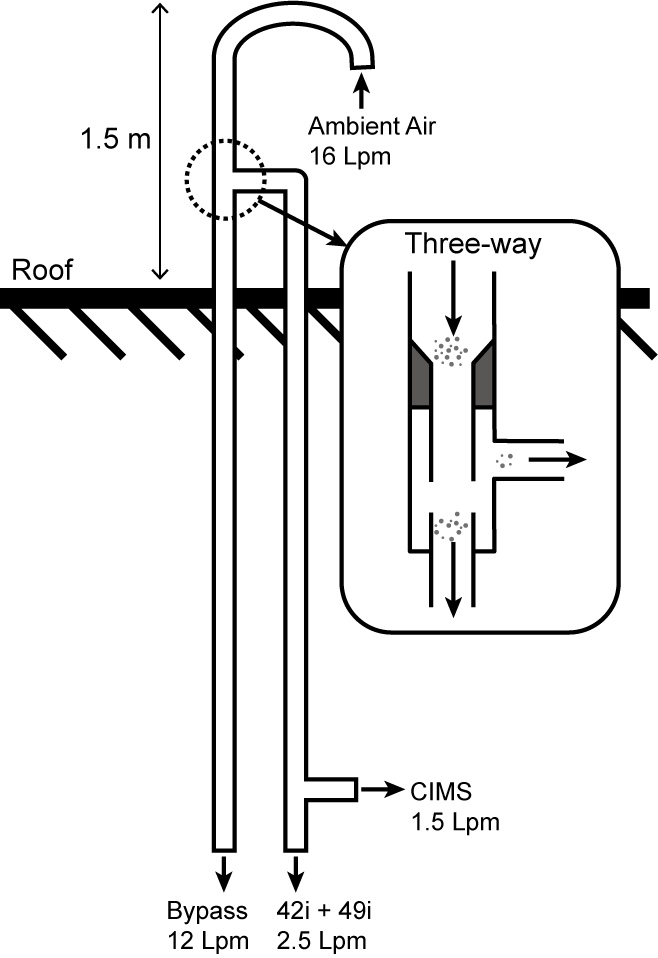


**Supplementary Figure 11:** **The schematic layout of the inlet configuration in the field study.** The structure of the insert was modified from a PFA three-way fitting. A diaphragm pump, which pulls 12 Lpm air, was adopted to divert large particles in the sampling air into the by-pass. The rest of the air is sucked into the CIMS, a NO_x_ analyzer (Model 42i, Thermo), and an O_3_ analyzer (Model 49i, Thermo) with a total flow of 4 LPM. The resident time from the outdoor inlet to the sampling point at CIMS was 0.5 seconds.


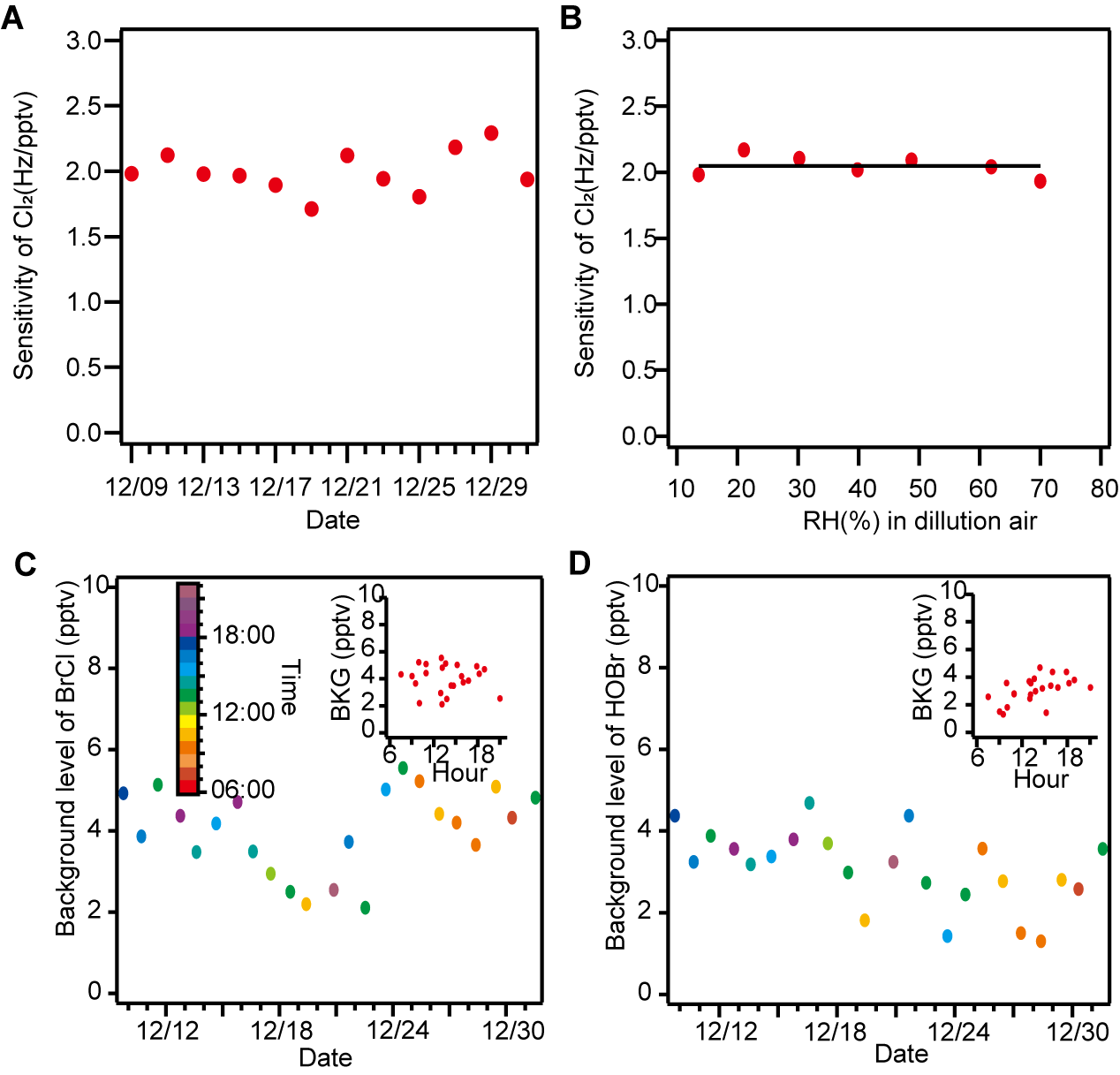


**Supplementary Figure 12: The CIMS instrumental sensitivity and background level (the signal equivalent to concentration) during the campaign. (A)**The sensitivity of Cl_2_ (with reagent ion IH_2_O^-^ at 145 amu normalized to 50k Hz) was calibrated every two days to confirm the stability of CIMS. **(B)**The sensitivity of Cl_2_ under different RH in dilution zero air. The RH-sensitivity relationship test was conducted on 9 Dec 2017 before ambient measurement. **(C)** The background level of BrCl during the campaign. **(D)** The background level of HOBr during the campaign. The background level was determined once per day. And the background tests were conducted at different periods during the campaign, and it did not show a significant daytime or night-time (right inset) bias.

**
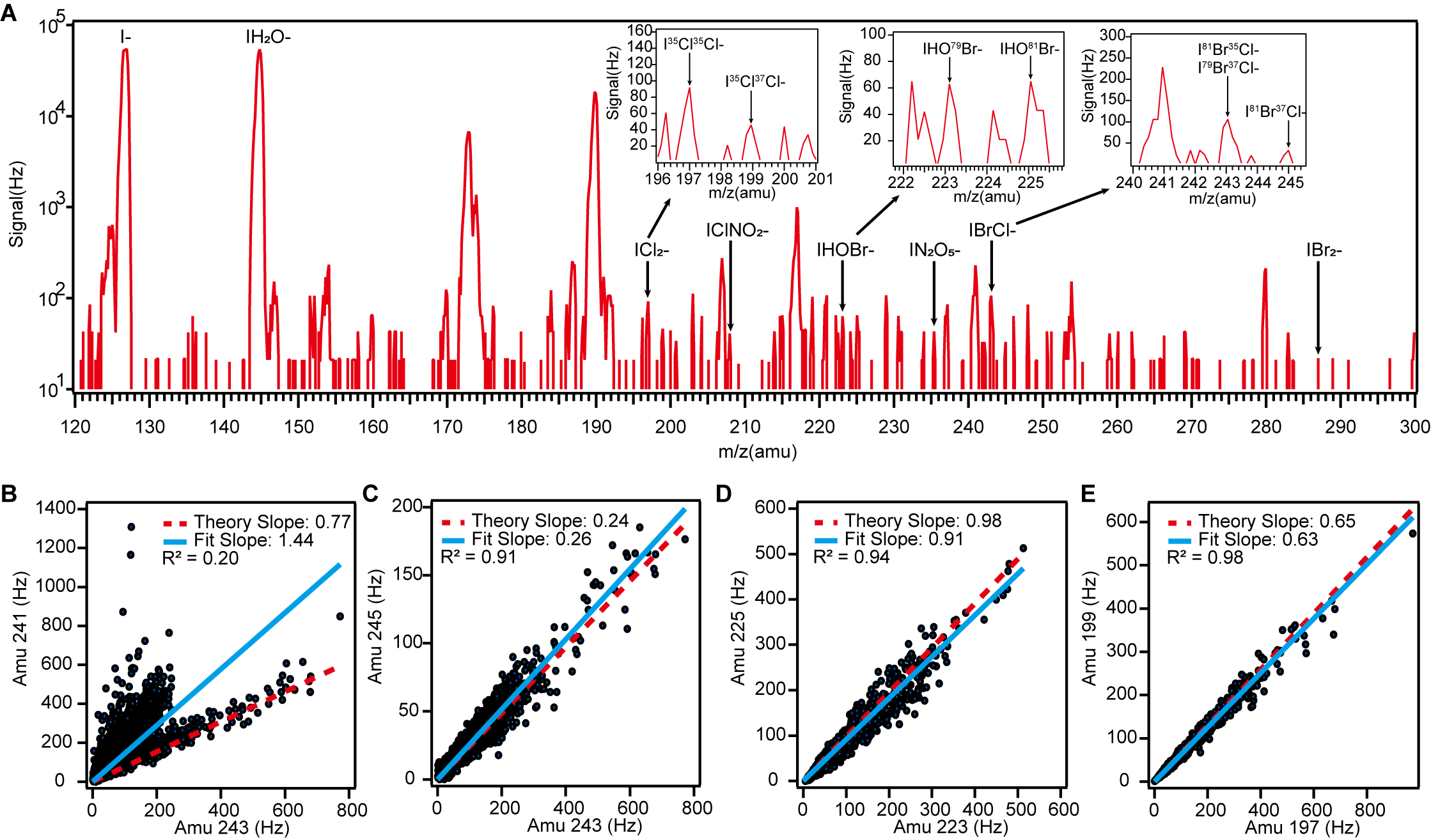
**

**Supplementary Figure 13: The mass spectrum and isotopic analysis of reactive halogen species for CIMS ambient measurement during 9-31 December 2017. (A)** An example of a mass spectrum for CIMS measurement from 120 amu to 300 amu during the field measurements in Wangdu. The signals below 10 Hz were not recorded during hourly scans but were recorded during measurements. The insert panels are the high-resolution scan spectra for Cl_2_, HOBr, and BrCl. **(B)** Scatter plot of the raw CIMS signal of BrCl at mass 243 amu (I^79^Br^37^Cl^-^; I^81^Br^35^Cl^-^) versus 241 amu (I^79^Br^35^Cl^-^) with 10 min average for the entire ambient measurement period during 9-31 December 2017. **(C)** Scatter plot of the raw CIMS signal of BrCl at mass 243 amu (I^79^Br^37^Cl^-^; I^81^Br^35^Cl^-^) versus 245 amu (I^81^Br^37^Cl^-^) with 10 min average for the entire ambient measurement period during 9-31 December 2017. **(D)** Scatter plot of the raw CIMS signal of HOBr at mass 223 amu (IHO^79^Br^-^) versus 225 amu (IHO^81^Br^-^) with 10 min average for the entire ambient measurement period during 9-31 December 2017. **(E)** Scatter plot of the raw CIMS signal of Cl_2_ at mass 199 amu (I^35^Cl^37^Cl^-^; I^37^Cl^35^Cl^-^)) versus 197 amu (I^35^Cl^35^Cl^-^) with 10 min average for the entire ambient measurement period during 9-31 December 2017. The blue lines are the measured ratios, and the red dashed lines are the theoretical isotopic ratios.


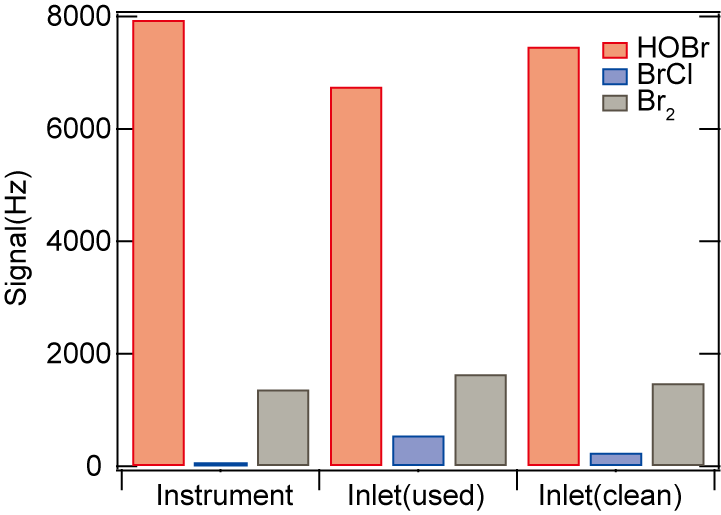


**Supplementary Figure 14: The measured signals of HOBr (orange bar), BrCl (blue bar), and Br_2_ (gray bar) when the synthesized HOBr mixed with humidified zero air was introduced to the instrument (CIMS) directly, passed through the field used Teflon inlet tubing, and passed through the new Teflon tubing with the same length.**

**
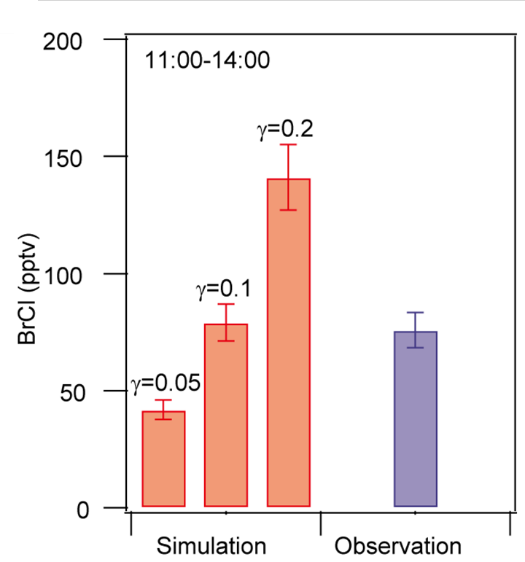
**

**Supplementary Figure 15: Comparison of the model simulated and observed average concentration of BrCl.** As the box model lacks sinks for BrCl during nighttime, we compared the simulation results with average observed concentration from 11:00 to 14:00. The model did not consider heterogeneous loss for BrCl in daytime as the photolysis was the dominant sink for BrCl.

**Supplementary Tables**

Supplementary Table 1: Coal supply and consumption in the top 20 countries/economics in 2017.

| Country/Economy | | Total coal supply (million tons)^1^ | Percentage of coal in total energy consumption (%)^2^ | Percentage of coal used for electricity (%)^1^ |
| --- | --- | --- | --- | --- |
| China | 2000 | | 64 | 55 |
| India | 384.3 | | 44 | 68 |
| United States | 335.1 | | 15 | 92 |
| Japan | 116.4 | | 27 | 62 |
| Russian Federation | 106.3 | | 16 | 56 |
| South Africa | 96.3 | | 74 | 59 |
| Korea | 83 | | 29 | 72 |
| Germany | 70.9 | | 23 | 79 |
| Poland | 50 | | 48 | 71 |
| Indonesia | 48.4 | | 20 | 77 |
| Australia | 43.8 | | 33 | 90 |
| Taiwan, Province of China | 41.5 | | 37 | 69 |
| Turkey | 39.1 | | 30 | 59 |
| Kazakhstan | 39 | | 45 | 57 |
| Viet Nam | 28.2 | | 35 | 48 |
| Ukraine | 25.8 | | 29 | 57 |
| Malaysia | 20.8 | | 25 | 91 |
| Canada | 17.3 | | 5 | 80 |
| Brazil | 16.8 | | 6 | 32 |
| Philippines | 16.5 | | 29 | 80 |

^1^ The data are directly from the International Energy Agency (IEA) [32].

^2^ The data are calculated using the coal consumption data divided by the total energy consumption which is the sum of coal, oil, natural gas, biofuels and waste, hydropower, wind and solar energy, and nuclear energy whose data are also from the IEA [32].

Supplementary Table 2: Chlorine and bromine reactions included in the box model.

| **Inorganic reactions** |  |  |  |
| --- | --- | --- | --- |
| **No.** | **Reaction** | **Rate constant** (cm^3^ molecule^-1^ s^-1^) at 298K, 1 atm | **Reference** |
| In.1 | Cl + O3 = ClO | 1.18E-11 | NASA |
| In.2 | Cl + HO2 = HCl + O2 | 3.46E-11 | NASA |
| In.3 | Cl + HO2 = ClO + OH | 1.02E-11 | IUPAC |
| In.4 | Cl + NO = NOCl | 2.40E-12 | NASA |
| In.5 | Cl + H2 = HCl | 1.68E-14 | IUPAC |
| In.6 | Cl + NO3 = ClO + NO2 | 2.40E-11 | IUPAC |
| In.7 | Cl + OClO = ClO + ClO | 5.82E-11 | IUPAC |
| In.8 | Cl + ClONO2 = Cl2 + NO3 | 1.02E-11 | NASA |
| In.9 | Cl + NO2 = ClNO2 | 3.84E-12 | NASA |
| In.10 | Cl + H2O2 = HCl + HO2 | 4.10E-13 | IUPAC |
| In.11 | Cl + HBr = HCl + Br | 5.88E-12 | Nicovich and Wine [33] |
| In.12 | ClO + O = Cl + O2 | 3.72E-11 | IUPAC |
| In.13 | ClO + HO2 = HOCl | 6.88E-12 | IUPAC |
| In.14 | ClO + ClO = Cl2 + O2 | 4.82E-15 | IUPAC |
| In.15 | ClO + ClO = Cl + ClOO | 8.06E-15 | IUPAC |
| In.16 | ClO + ClO = OClO + Cl | 3.53E-15 | IUPAC |
| In.17 | ClO + ClO = Cl2O2 | 4.26E-13 | IUPAC |
| In.18 | Cl2O2 = ClO + ClO | 4.49E+01 | IUPAC |
| In.19 | ClO + NO = Cl + NO2 | 1.69E-11 | IUPAC |
| In.20 | ClO + NO2 = ClONO2 | 2.46E-12 | NASA |
| In.21 | ClO + NO3 = ClOO + NO2 | 3.40E-13 | IUPAC |
| In.22 | ClO + NO3 = OClO + NO2 | 1.20E-13 | IUPAC |
| In.23 | ClO + OH = Cl + HO2 | 1.83E-11 | IUPAC |
| In.24 | ClO + OH = HCl + O2 | 1.30E-12 | IUPAC |
| In.25 | ClO + BrO = Br + OClO | 6.02E-12 | NASA |
| In.26 | ClO + BrO = Br + ClOO | 5.50E-12 | NASA |
| In.27 | ClO + BrO = BrCl | 1.08E-12 | NASA |
| In.28 | ClO + CH3O2 = Cl + HCHO + HO2 | 2.40E-13 | NASA |
| In.29 | ClO + CH3CO3 = Cl + CH3O2 | 2.03E-12 | Michalowski et al. [34] |
| In.30 | OClO + OH = HOCl + O2 | 1.05E-11 | NASA |
| In.31 | OClO + NO = ClO + NO2 | 3.34E-13 | NASA |
| In.32 | OClO + O = ClO | 9.58E-14 | IUPAC |
| In.33 | HOCl + OH = ClO + H2O | 5.60E-13 | NASA |
| In.34 | HOCl + O = OH +ClO | 1.70E-13 | IUPAC |
| In.35 | HCl + OH = Cl + H2O | 7.78E-13 | NASA |
| In.36 | OH + ClNO2 = HOCl + NO2 | 3.62E-14 | NASA |
| In.37 | NOCl + H2O = HCl + HONO | 4.57E-05 | NIST |
| In.38 | ClONO2 + OH = HOCl + NO3 | 1.98E-13 | NASA |
| In.39 | ClONO2 + OH = HNO3 +ClO | 1.98E-13 | NASA |
| In.40 | ClONO2 + O = ClO + NO3 | 2.15E-13 | IUPAC |
| In.41 | ClONO2 +H2O = HOCl + HNO3 | 3.08E-04 | NIST |
| In.42 | Cl2 + OH = HOCl + Cl | 6.48E-14 | NASA |
| In.43 | Br + O3 = BrO | 1.16E-12 | NASA |
| In.44 | Br2 + OH= HOBr + Br | 4.48E-11 | NASA |
| In.45 | Br + HO2 = HBr | 1.70E-12 | NASA |
| In.46 | Br + NO2 = BrNO2 | 5.11E-13 | NASA |
| In.47 | Br + BrNO3 = Br2 + NO3 | 6.04E-11 | NIST |
| In.48 | Br + OClO = BrO + ClO | 3.44E-13 | NASA |
| In.49 | Br + NO3 = BrO + NO2 | 1.60E-11 | NASA |
| In.50 | BrO + O = Br | 4.11E-11 | NASA |
| In.51 | BrO + OH = Br + HO2 | 4.16E-11 | NASA |
| In.52 | BrO + HO2 = HOBr | 2.41E-11 | NASA |
| In.53 | BrO + CH3O2 = HOBr + HCOOH | 4.55E-12 | IUPAC |
| In.54 | BrO + CH3O2 = Br + HCHO + HO2 | 1.14E-12 | IUPAC |
| In.55 | BrO + CH3CO3 = Br + CH3O2 | 1.70E-12 | Michalowski et al. [34] |
| In.56 | BrO + C2H5CHO = HOBr + C2H5CO3 | 1.50E-14 | Michalowski et al. [34] |
| In.57 | BrO + NO = Br + NO2 | 2.08E-11 | NASA |
| In.58 | BrO + NO2 = BrNO3 | 2.89E-12 | NASA |
| In.59 | BrO + BrO = Br + Br | 2.70E-12 | NASA |
| In.60 | BrO + BrO = Br2 | 4.86E-13 | NASA |
| In.61 | BrO + HBr = HOBr + Br | 2.10E-14 | Michalowski et al. [34] |
| In.62 | HBr + OH = Br | 1.13E-11 | NASA |
| In.63 | Cl + BrCl = Br + Cl2 | 1.50E-11 | NIST |
| In.64 | Br + Cl2 = BrCl + Cl | 1.10E-15 | NIST |
| In.65 | Cl + Br2 = BrCl + Br | 1.94E-10 | NIST |
| In.66 | Br + BrCl = Br2 + Cl | 3.30E-15 | NIST |
| In.67 | HOBr + O --> BrO + OH | 2.83E-11 | NASA |
| In.68 | HOBr + OH = BrO | 5.00E-13 | NIST |
| In.69 | HOBr + Cl = BrCl + OH | 8.00E-11 | NIST |
| **Cl + VOCs** |  |  |  |
| **No.** | **Reaction** | **Rate constant** (cm^3^ molecule^-1^ s^-1^) at 298K, 1 atm | **Reference** |
| OrCl.1 | C2H5O2 + Cl --> C2H5O + ClO | 7.40E-11 | NASA |
| OrCl.2 | HCHO + Cl --> HCl + CO + HO2 | 7.23E-11 | NASA |
| OrCl.3 | CH3CHO + Cl --> HCl + CH3CO3 | 7.92E-11 | IUPAC |
| OrCl.4 | CH3CHO + Cl --> HCl + HCOCH2O2 | 8.00E-13 | IUPAC |
| OrCl.5 | C2H5CHO + Cl --> HCl + C2H5CO3 | 1.30E-10 | IUPAC |
| OrCl.6 | C3H7CHO + Cl --> BUTALO2 + HCl | 2.08E-11 | NIST; Branch ratio refer to OH |
| OrCl.7 | C3H7CHO + Cl --> HCl + C3H7CO3 | 1.17E-10 | NIST; Branch ratio refer to OH |
| OrCl.8 | IPRCHO + Cl --> IBUTALBO2 + HCl | 9.34E-12 | NIST; Branch ratio refer to OH |
| OrCl.9 | IPRCHO + Cl --> IBUTALCO2 + HCl | 1.02E-11 | NIST; Branch ratio refer to OH |
| OrCl.10 | IPRCHO + Cl --> HCl + IPRCO3 | 1.53E-10 | NIST; Branch ratio refer to OH |
| OrCl.11 | C4H9CHO + Cl --> C4CHOBO2 + HCl | 3.59E-11 | NIST; Branch ratio refer to OH |
| OrCl.12 | C4H9CHO + Cl --> C4H9CO3 + HCl | 1.53E-10 | NIST; Branch ratio refer to OH |
| OrCl.13 | BENZAL + Cl --> C6H5CO3 + HCl | 8.97E-11 | NIST |
| OrCl.14 | GLYOX + Cl --> HCl + 2 CO + HO2 | 4.34E-11 | NIST; Branch ratio refer to OH |
| OrCl.15 | GLYOX + Cl --> HCl + HCOCO3 | 2.89E-11 | NIST; Branch ratio refer to OH |
| OrCl.16 | MGLYOX + Cl --> HCl + CO + CH3CO3 | 8.00E-11 | NIST |
| OrCl.17 | MACR + Cl --> HCl + MACO3 | 4.70E-11 | NIST |
| OrCl.18 | CH3COCH3 + Cl --> HCl + CH3COCH2O2 | 2.10E-12 | IUPAC |
| OrCl.19 | MEK + Cl --> HCl + MEKAO2 | 1.83E-11 | NIST; Branch ratio refer to OH |
| OrCl.20 | MEK + Cl --> HCl + MEKBO2 | 1.84E-11 | NIST; Branch ratio refer to OH |
| OrCl.21 | MEK + Cl --> HCl + MEKCO2 | 3.15E-12 | NIST; Branch ratio refer to OH |
| OrCl.22 | MPRK + Cl --> HCl + CO2C54O2 | 3.41E-11 | NIST; Branch ratio refer to OH |
| OrCl.23 | MPRK + Cl --> MPRKAO2 + HCl | 7.59E-12 | NIST; Branch ratio refer to OH |
| OrCl.24 | DIEK + Cl --> DIEKAO2 + HCl | 3.80E-11 | NIST; Branch ratio refer to OH |
| OrCl.25 | DIEK + Cl --> HCl + DIEKBO2 | 3.78E-11 | NIST; Branch ratio refer to OH |
| OrCl.26 | MIPK + Cl --> MIPKAO2 + HCl | 2.28E-11 | NIST; Branch ratio refer to OH |
| OrCl.27 | MIPK + Cl --> MIPKBO2 + HCl | 2.08E-11 | NIST; Branch ratio refer to OH |
| OrCl.28 | HEX2ONE + Cl --> HEX2ONAO2 + HCl | 4.75E-11 | NIST; Branch ratio refer to OH |
| OrCl.29 | HEX2ONE + Cl --> HEX2ONBO2 + HCl | 1.08E-11 | NIST; Branch ratio refer to OH |
| OrCl.30 | HEX2ONE + Cl --> HEX2ONCO2 + HCl | 8.17E-12 | NIST; Branch ratio refer to OH |
| OrCl.31 | HEX3ONE + Cl --> HEX3ONAO2 + HCl | 5.26E-11 | NIST; Branch ratio refer to OH |
| OrCl.32 | HEX3ONE + Cl --> HEX3ONBO2 + HCl | 1.17E-11 | NIST; Branch ratio refer to OH |
| OrCl.33 | HEX3ONE + Cl --> HEX3ONCO2 + HCl | 9.06E-12 | NIST; Branch ratio refer to OH |
| OrCl.34 | HEX3ONE + Cl --> HEX3ONDO2 + HCl | 9.06E-12 | NIST; Branch ratio refer to OH |
| OrCl.35 | MIBK + Cl --> MIBKAO2 + HCl | 7.68E-11 | NIST; Branch ratio refer to OH |
| OrCl.36 | MIBK + Cl --> MIBKBO2 + HCl | 7.59E-12 | NIST; Branch ratio refer to OH |
| OrCl.37 | MTBK + Cl --> MTBKO2 + HCl | 8.44E-11 | refer to MIBK |
| OrCl.38 | CYHEXONE + Cl --> CYHXONAO2 + HCl | 1.26E-10 | NIST |
| OrCl.39 | CH3OH + Cl --> HCl + HCHO + HO2 | 5.52E-11 | NASA |
| OrCl.40 | C2H5OH + Cl --> HCl + CH3CHO + HO2 | 9.29E-11 | NASA |
| OrCl.41 | C2H5OH + Cl --> HCl + HOCH2CH2O2 | 8.07E-12 | NASA |
| OrCl.42 | NPROPOL + Cl --> HCl + C2H5CHO + HO2 | 9.43E-11 | IUPAC |
| OrCl.43 | NPROPOL + Cl --> HO1C3O2 + HCl | 2.36E-11 | IUPAC |
| OrCl.44 | NPROPOL + Cl --> HCl + HYPROPO2 | 3.93E-11 | IUPAC |
| OrCl.45 | IPROPOL + Cl --> HCl + CH3COCH3 + HO2 | 7.40E-11 | IUPAC |
| OrCl.46 | IPROPOL + Cl --> HCl + IPROPOLO2 | 1.30E-11 | IUPAC |
| OrCl.47 | NBUTOL + Cl --> HCl + C3H7CHO + HO2 | 7.93E-11 | IUPAC; Branch ratio refer to OH |
| OrCl.48 | NBUTOL + Cl --> NBUTOLAO2 + HCl | 7.11E-11 | IUPAC; Branch ratio refer to OH |
| OrCl.49 | NBUTOL + Cl --> NBUTOLBO2 + HCl | 7.11E-11 | IUPAC; Branch ratio refer to OH |
| OrCl.50 | BUT2OL + Cl --> BUT2OLO2 + HCl | 3.99E-11 | NIST; Branch ratio refer to OH |
| OrCl.51 | BUT2OL + Cl --> MEK + HCl + HO2 | 7.06E-11 | NIST; Branch ratio refer to OH |
| OrCl.52 | IBUTOL + Cl --> IBUTOLBO2 + HCl | 1.02E-10 | NIST; Branch ratio refer to OH |
| OrCl.53 | IBUTOL + Cl --> IBUTOLCO2 + HCl | 1.64E-11 | NIST; Branch ratio refer to OH |
| OrCl.54 | IBUTOL + Cl --> HCl + IPRCHO + HO2 | 6.41E-11 | NIST; Branch ratio refer to OH |
| OrCl.55 | TBUTOL + Cl --> TBUTOLO2 + HCl | 2.80E-11 | NIST; Branch ratio refer to OH |
| OrCl.56 | TBUTOL + Cl --> TC4H9O + HCl | 3.53E-12 | NIST; Branch ratio refer to OH |
| OrCl.57 | PECOH + Cl --> DIEK + HCl + HO2 | 8.89E-11 | NIST; Branch ratio refer to OH |
| OrCl.58 | PECOH + Cl --> HCl + HO3C5O2 | 1.43E-11 | NIST; Branch ratio refer to OH |
| OrCl.59 | PECOH + Cl --> PE2ENEBO2 + HCl | 1.01E-10 | NIST; Branch ratio refer to OH |
| OrCl.60 | IPEAOH + Cl --> BUT2CHO + HCl + HO2 | 5.52E-11 | NIST; Branch ratio refer to OH |
| OrCl.61 | IPEAOH + Cl --> HM2C43O2 + HCl | 4.94E-11 | NIST; Branch ratio refer to OH |
| OrCl.62 | IPEAOH + Cl --> M2BUOL2O2 + HCl | 8.70E-11 | NIST; Branch ratio refer to OH |
| OrCl.63 | IPECOH + Cl --> HCl + HO2M2C4O2 | 7.46E-12 | NIST; Branch ratio refer to OH |
| OrCl.64 | IPECOH + Cl --> ME2BU2OLO2 + HCl | 5.23E-11 | NIST; Branch ratio refer to OH |
| OrCl.65 | IPECOH + Cl --> PROL11MO2 + HCl | 1.48E-11 | NIST; Branch ratio refer to OH |
| OrCl.66 | IPEBOH + Cl --> H2M3C4O2 + HCl | 8.87E-12 | NIST; Branch ratio refer to OH |
| OrCl.67 | IPEBOH + Cl --> ME2BUOLO2 + HCl | 5.55E-11 | NIST; Branch ratio refer to OH |
| OrCl.68 | IPEBOH + Cl --> MIPK + HCl + HO2 | 5.55E-11 | NIST; Branch ratio refer to OH |
| OrCl.69 | CYHEXOL + Cl --> CYHEXOLAO2 + HCl | 2.37E-10 | NIST; Branch ratio refer to OH |
| OrCl.70 | CYHEXOL + Cl --> CYHEXONE + HCl + HO2 | 8.38E-11 | NIST; Branch ratio refer to OH |
| OrCl.71 | MIBKAOH + Cl --> MIBKAOHAO2 + HCl | 3.41E-11 | NIST; Branch ratio refer to OH |
| OrCl.72 | MIBKAOH + Cl --> MIBKAOHBO2 + HCl | 1.33E-11 | NIST; Branch ratio refer to OH |
| OrCl.73 | MIBKAOH + Cl --> MIBKHO4O2 + HCl | 1.82E-12 | NIST; Branch ratio refer to OH |
| OrCl.74 | ETHGLY + Cl --> HCl + HOCH2CHO + HO2 | 2.48E-10 | NIST |
| OrCl.75 | PROPGLY + Cl --> HCl + ACETOL + HO2 | 1.26E-10 | NIST; Branch ratio refer to OH |
| OrCl.76 | PROPGLY + Cl --> CH3CHOHCHO + HCl + HO2 | 7.94E-11 | NIST; Branch ratio refer to OH |
| OrCl.77 | CRESOL + Cl --> OXYL1O2 + HCl | 6.20E-11 | Refer to kCl/kOH ratio of average of EThGLY and PROPGLY; |
| OrCl.78 | CH3OOH + Cl --> HCl + CH3O2 | 3.54E-11 | IUPAC |
| OrCl.79 | CH3OOH + Cl --> HCl + HCHO + OH | 2.36E-11 | IUPAC |
| OrCl.80 | HCOOH + Cl --> HCl + HO2 | 1.90E-13 | IUPAC |
| OrCl.81 | CH3CO2H + Cl --> HCl + CH3O2 | 2.65E-14 | IUPAC |
| OrCl.82 | PROPACID + Cl --> HCl + C2H5O2 | 3.96E-14 | Refer to kCl/kOH ratio of average of HCOOH and CH3CO2H; |
| OrCl.83 | CH3NO3 + Cl --> HCl + HCHO + NO2 | 2.40E-13 | IUPAC |
| OrCl.84 | C2H5NO3 + Cl --> HCl + CH3CHO + NO2 | 4.70E-12 | IUPAC |
| OrCl.85 | NC3H7NO3 + Cl --> HCl + C2H5CHO + NO2 | 2.20E-11 | IUPAC |
| OrCl.86 | IC3H7NO3 + Cl --> HCl + CH3COCH3 + NO2 | 3.80E-12 | IUPAC |
| OrCl.87 | NC4H9NO3 + Cl --> HCl + C3H7CHO + NO2 | 8.50E-11 | IUPAC |
| OrCl.88 | TOLUENE + Cl --> C6H5CH2O2 + HCl | 5.90E-11 | NIST |
| OrCl.89 | OXYL + Cl --> OXYLO2 + HCl | 1.50E-10 | NIST |
| OrCl.90 | MXYL + Cl --> MXYLO2 + HCl | 1.71E-10 | NIST |
| OrCl.91 | PXYL + Cl --> PXYLO2 + HCl | 2.65E-10 | NIST |
| OrCl.92 | EBENZ + Cl --> C6H5C2H4O2 + HCl | 9.07E-11 | NIST |
| OrCl.93 | PBENZ + Cl --> PHC3O2 + HCl | 6.98E-11 | NIST |
| OrCl.94 | IPBENZ + Cl --> PHIC3O2 + HCl | 7.59E-11 | NIST |
| OrCl.95 | TM123B + Cl --> TM123BO2 + HCl | 3.37E-10 | NIST |
| OrCl.96 | TM124B + Cl --> TM124BO2 + HCl | 3.35E-10 | NIST |
| OrCl.97 | TM135B + Cl --> TMBO2 + HCl | 2.93E-10 | NIST |
| OrCl.98 | OETHTOL + Cl --> ETOLO2 + HCl | 1.02E-10 | NIST |
| OrCl.99 | METHTOL + Cl --> ETOLO2 + HCl | 1.28E-10 | NIST |
| OrCl.100 | PETHTOL + Cl --> ETOLO2 + HCl | 2.03E-10 | NIST |
| OrCl.101 | C2H4 + Cl --> CH2ClCH2O2 | 1.00E-10 | IUPAC |
| OrCl.102 | C3H6 + Cl --> C3H5O2 + HCl | 2.70E-11 | IUPAC |
| OrCl.103 | C3H6 + Cl --> IPROClO2 | 1.35E-10 | IUPAC |
| OrCl.104 | C3H6 + Cl --> HYPROClO2 | 1.08E-10 | IUPAC |
| OrCl.105 | C3H5O2 + NO --> ACR + NO2 + HO2 | 9.04E-12 | Xue et al. [23] |
| OrCl.106 | C3H5O2 + HO2 --> PROPACID | 1.19E-11 | Xue et al. [23] |
| OrCl.107 | C3H5O2 + NO3 --> ACR + NO2 + HO2 | 2.30E-12 | Xue et al. [23] |
| OrCl.108 | C3H5O2 --> ACR + HO2 | 7.22E-08 | Xue et al. [23] |
| OrCl.109 | C3H5O2 --> C3H5O2H | 4.81E-08 | Xue et al. [23] |
| OrCl.110 | IPROClO2 + HO2 --> IPROClO2H | 1.19E-11 | Xue et al. [23] |
| OrCl.111 | IPROClO2 + NO --> CH3CHClCHO + NO2 + HO2 | 9.04E-12 | Xue et al. [23] |
| OrCl.112 | IPROClO2 + NO3 --> CH3CHClCHO + NO2 + HO2 | 2.30E-12 | Xue et al. [23] |
| OrCl.113 | IPROClO2 --> CH3CHClCHO + HO2 | 7.22E-08 | Xue et al. [23] |
| OrCl.114 | IPROClO2 --> IPROClO2H | 4.81E-08 | Xue et al. [23] |
| OrCl.115 | CH3CHClCHO + NO3 --> CH3CHClCO3 + HNO3 | 6.54E-15 | Xue et al. [23] |
| OrCl.116 | CH3CHClCHO + OH --> CH3CHClCO3 | 1.70E-11 | Xue et al. [23] |
| OrCl.117 | CH3CHClCHO --> CH3CHClO2 + CO + HO2 | 2.00E-12*RO2 | Xue et al. [23] |
| OrCl.118 | CH3CHClCO3 + HO2 --> CH3CHClO2 + OH | 6.13E-12 | Xue et al. [23] |
| OrCl.119 | CH3CHClCO3 + HO2 --> IPROClPER | 5.71E-12 | Xue et al. [23] |
| OrCl.120 | CH3CHClCO3 + HO2 --> C2H4ClCO2H + O3 | 2.09E-12 | Xue et al. [23] |
| OrCl.121 | CH3CHClCO3 + NO --> CH3CHClO2 + NO2 | 1.98E-11 | Xue et al. [23] |
| OrCl.122 | CH3CHClCO3 + NO2 --> IPROClPAN | 8.99E-12 | Xue et al. [23] |
| OrCl.123 | CH3CHClCO3 + NO3 --> CH3CHClO2 + NO2 | 4.00E-12 | Xue et al. [23] |
| OrCl.124 | CH3CHClCO3 --> CH3CHClO2 | 4.21E-07 | Xue et al. [23] |
| OrCl.125 | CH3CHClCO3 --> C2H4ClCO2H | 1.80E-07 | Xue et al. [23] |
| OrCl.126 | IPROClPER + OH --> CH3CHClCO3 | 9.34E-12 | Xue et al. [23] |
| OrCl.127 | IPROClPER --> CH3CHClO2 + OH | 2.00E-12*RO2 | Xue et al. [23] |
| OrCl.128 | C2H4ClCO2H + OH --> CH3CHClO2 | 1.20E-12 | Xue et al. [23] |
| OrCl.129 | IPROClPAN + OH --> ClETAL + NO2 + CO | 2.34E-12 | Xue et al. [23] |
| OrCl.130 | IPROClPAN --> CH3CHClCO3 + NO2 | 4.32E-04 | Xue et al. [23] |
| OrCl.131 | HYPROClO2 + HO2 --> HYPROClO2H | 1.19E-11 | Xue et al. [23] |
| OrCl.132 | HYPROClO2 + NO3 --> CH3CHClCHO + NO2 + HO2 | 2.30E-12 | Xue et al. [23] |
| OrCl.133 | HYPROClO2 --> CH3CHClCHO + HO2 | 3.18E-08 | Xue et al. [23] |
| OrCl.134 | HYPROClO2 --> HYPROClO2H | 2.12E-08 | Xue et al. [23] |
| OrCl.135 | HYPROClO2 + NO --> CH3CHClCHO + NO2 + HO2 | 9.04E-12 | Xue et al. [23] |
| OrCl.136 | BUT1ENE + Cl --> OLEClO2 | 2.91E-10 | NIST |
| OrCl.137 | CBUT2ENE + Cl --> OLEClO2 | 3.58E-10 | NIST |
| OrCl.138 | TBUT2ENE + Cl --> OLEClO2 | 3.58E-10 | NIST |
| OrCl.139 | MEPROPENE + Cl --> OLEClO2 | 1.42E-10 | NIST |
| OrCl.140 | PENT1ENE + Cl --> OLEClO2 | 1.66E-10 | NIST |
| OrCl.141 | CPENT2ENE + Cl --> OLEClO2 | 1.66E-10 | NIST |
| OrCl.142 | TPENT2ENE + Cl --> OLEClO2 | 1.66E-10 | NIST |
| OrCl.143 | ME2BUT1ENE + Cl --> OLEClO2 | 3.58E-10 | NIST |
| OrCl.144 | ME3BUT1ENE + Cl --> OLEClO2 | 3.29E-10 | NIST |
| OrCl.145 | ME2BUT2ENE + Cl --> OLEClO2 | 3.95E-10 | NIST |
| OrCl.146 | HEX1ENE + Cl --> OLEClO2 | 4.00E-10 | NIST |
| OrCl.147 | CHEX2ENE + Cl --> OLEClO2 | 4.26E-10 | NIST |
| OrCl.148 | THEX2ENE + Cl --> OLEClO2 | 4.26E-10 | NIST |
| OrCl.149 | DM23BU2ENE + Cl --> OLEClO2 | 7.81E-10 | NIST |
| OrCl.150 | STYRENE + Cl --> OLEClO2 | 3.60E-10 | NIST |
| OrCl.151 | MVK + Cl --> OLEClO2 | 2.20E-10 | NIST |
| OrCl.152 | MACR + Cl --> OLEClO2 | 1.70E-10 | NIST |
| OrCl.153 | OLEClO2 + HO2 = OLECLO2H | 1.61E-11 | Xue et al. [23] |
| OrCl.154 | OLEClO2 + NO --> OLEClCHO + NO2 + HO2 | 9.04E-12 | Xue et al. [23] |
| OrCl.155 | OLEClO2 + NO3 --> OLEClCHO + NO2 + HO2 | 2.30E-12 | Xue et al. [23] |
| OrCl.156 | OLEClO2 --> OLEClCHO + HO2 | 7.22E-08 | Xue et al. [23] |
| OrCl.157 | OLEClO2 = DUMMY | 4.81E-08 | Xue et al. [23] |
| OrCl.158 | OLEClCHO + NO3 --> OLEClCO3 + HNO3 | 1.50E-14 | Xue et al. [23] |
| OrCl.159 | OLEClCHO + OH --> OLEClCO3 | 2.85E-11 | Xue et al. [23] |
| OrCl.160 | OLEClCHO --> OLEClO2 + CO + HO2 | 2.00E-12*RO2 | Xue et al. [23] |
| OrCl.161 | OLEClCO3 + HO2 = DUMMY | 1.39E-11 | Xue et al. [23] |
| OrCl.162 | OLEClCO3 + NO --> OLEClO2 + NO2 | 1.98E-11 | Xue et al. [23] |
| OrCl.163 | OLEClCO3 + NO3 --> OLEClO2 + NO2 | 4.00E-12 | Xue et al. [23] |
| OrCl.164 | OLEClCO3 --> OLEClO2 | 4.21E-07 | Xue et al. [23] |
| OrCl.165 | OLEClCO3 --> HALODUMMY | 1.80E-07 | Xue et al. [23] |
| OrCl.166 | OLEClCO3 + NO2 --> OLEClPAN | 8.99E-12 | Xue et al. [23] |
| OrCl.167 | OLEClPAN --> OLEClCO3 + NO2 | 4.32E-04 | Xue et al. [23] |
| OrCl.168 | C5H8 + Cl --> ISOClO2 | 4.75E-10 | Xue et al. [23] |
| OrCl.169 | ISOClO2 + NO --> ISOClO + NO2 | 9.04E-12 | Xue et al. [23] |
| OrCl.170 | ISOClO = ISOCLCHO + CH3O2 | 1.00E+06 | Xue et al. [23] |
| OrCl.171 | C2H2 + Cl --> CHOCl + CO + HO2 | 4.97E-11 | Xue et al. [23] |
| OrCl.172 | DMS + Cl --> CH3SCH2O2 + HCl | 3.30E-10 | Sander et al. [35] |
| OrCl.173 | DMS + Br --> CH3SCH2O2 + HBr | 3.00E-14 | Sander et al. [35] |
| OrCl.174 | DMS + BrO --> DMSO + Br | 4.40E-13 | Sander et al. [35] |
| OrCl.175 | ACR + Cl --> ACRO2 + HCl | 3.82E-11 | NIST; Branch ratio refer to OH |
| OrCl.176 | ACR + Cl --> OLEClO2 | 1.80E-11 | NIST; Branch ratio refer to OH |
| OrCl.177 | C4ALDB + Cl --> C3DBCO3 + HCl | 1.25E-10 | NIST; Branch ratio refer to OH |
| OrCl.178 | C4ALDB + Cl --> OLEBrO2 | 1.25E-10 | NIST; Branch ratio refer to OH |
| OrCl.179 | ME3BUOL + Cl --> C3ME3CHO + HCl + HO2 | 6.83E-11 | NIST; Branch ratio refer to OH |
| OrCl.180 | ME3BUOL + Cl --> HM33C3O2 + HCl | 1.08E-10 | NIST; Branch ratio refer to OH |
| OrCl.181 | ME3BUOL + Cl --> ME3BUOLO2 + HCl | 6.11E-11 | NIST; Branch ratio refer to OH |
| OrCl.182 | MBO + Cl --> MBOAO2 + HCl | 2.21E-10 | NIST; Branch ratio refer to OH |
| OrCl.183 | MBO + Cl --> MBOBO2 + HCl | 2.21E-10 | NIST; Branch ratio refer to OH |
| OrCl.184 | ACETOL + Cl --> HCl + MGLYOX | 5.60E-11 | NIST |
| OrCl.185 | CH3Cl + Cl --> CH2ClO2 + HCl | 4.96E-13 | NIST |
| OrCl.186 | CH2Cl2 + Cl --> CHCl2O2 + HCl | 3.58E-13 | NIST |
| OrCl.187 | CHCl3 + Cl --> CCl3O2 + HCl | 7.67E-14 | NIST |
| OrCl.188 | CH3CCl3 + Cl --> CCl3CH2O2 + HCl | 6.87E-15 | NIST |
| OrCl.189 | TRIClETH + Cl --> CHCl2Cl2O2 | 8.70E-11 | NIST |
| OrCl.190 | CDIClETH + Cl --> CHCl3O2 | 1.22E-10 | NIST |
| OrCl.191 | TDIClETH + Cl --> CHCl3O2 | 9.80E-11 | NIST |
| OrCl.192 | CH2ClCH2Cl + Cl --> DIClETO2 + HCl | 1.27E-12 | NIST |
| OrCl.193 | CCl2CH2 + Cl --> CH2Cl3O2 | 1.32E-10 | NIST |
| OrCl.194 | Cl12PROP + Cl --> Cl12PRAO2 + HCl | 4.29E-13 | NIST; Branch ratio refer to OH |
| OrCl.195 | Cl12PROP + Cl --> Cl12PRBO2 + HCl | 2.34E-12 | NIST; Branch ratio refer to OH |
| OrCl.196 | Cl12PROP + Cl --> Cl12PRCO2 + HCl | 1.13E-12 | NIST; Branch ratio refer to OH |
| OrCl.197 | CHCl2CH3 + Cl --> CH3CCl2O2 + HCl | 1.20E-12 | NIST; Branch ratio refer to OH |
| OrCl.198 | CHCl2CH3 + Cl --> CHCl2CH2O2 + HCl | 7.66E-14 | NIST; Branch ratio refer to OH |
| OrCl.199 | CH3CH2Cl + Cl --> CH3CHClO2 + HCl | 6.26E-12 | NIST; Branch ratio refer to OH |
| OrCl.200 | CH3CH2Cl + Cl --> CH2ClCH2O2 + HCl | 2.09E-12 | NIST; Branch ratio refer to OH |
| OrCl.201 | CHCl2CHCl2 + Cl --> CHCl2Cl2O2 + HCl | 1.91E-13 | NIST |
| OrCl.202 | CH2ClCHCl2 + Cl --> CH2Cl3O2 + HCl | 1.75E-13 | NIST; Branch ratio refer to OH |
| OrCl.203 | CH2ClCHCl2 + Cl --> CHCl3O2 + HCl | 1.75E-13 | NIST; Branch ratio refer to OH |
| OrCl.204 | VINCl + Cl --> CHCl2CH2O2 | 5.85E-11 | NIST; Branch ratio refer to OH |
| OrCl.205 | VINCl + Cl --> DIClETO2 | 5.85E-11 | NIST; Branch ratio refer to OH |
| OrCl.206 | C4H6 + Cl --> OLEClO2 | 4.20E-10 | NIST |
| OrCl.207 | CH3OCHO + Cl --> CHOOCH2O2 + HCl | 7.15E-13 | NIST; Branch ratio refer to OH |
| OrCl.208 | CH3OCHO + Cl --> HCl + CH3O2 | 5.85E-13 | NIST; Branch ratio refer to OH |
| OrCl.209 | METHACET + Cl --> METHACETO2 + HCl | 1.91E-12 | NIST; Branch ratio refer to OH |
| OrCl.210 | METHACET + Cl --> MOCOCH2O2 + HCl | 8.79E-13 | NIST; Branch ratio refer to OH |
| OrCl.211 | ETHACET + Cl --> ETHACETO2 + HCl | 1.16E-11 | NIST; Branch ratio refer to OH |
| OrCl.212 | ETHACET + Cl --> EOCOCH2O2 + HCl | 7.81E-13 | NIST; Branch ratio refer to OH |
| OrCl.213 | ETHACET + Cl --> ACETC2H4O2 + HCl | 1.30E-12 | NIST; Branch ratio refer to OH |
| OrCl.214 | NPROACET + Cl --> NPROACEAO2 + HCl | 1.67E-11 | NIST; Branch ratio refer to OH |
| OrCl.215 | NPROACET + Cl --> NPROACEBO2 + HCl | 2.68E-11 | NIST; Branch ratio refer to OH |
| OrCl.216 | NPROACET + Cl --> NPROACECO2 + HCl | 2.44E-12 | NIST; Branch ratio refer to OH |
| OrCl.217 | IPROACET + Cl --> IPROACETO2 + HCl | 2.67E-11 | NIST |
| OrCl.218 | NBUTACET + Cl --> NBUACETAO2 + HCl | 3.21E-11 | NIST; Branch ratio refer to OH |
| OrCl.219 | NBUTACET + Cl --> NBUACETBO2 + HCl | 3.95E-11 | NIST; Branch ratio refer to OH |
| OrCl.220 | NBUTACET + Cl --> NBUACETCO2 + HCl | 5.13E-11 | NIST; Branch ratio refer to OH |
| OrCl.221 | SBUTACET + Cl --> SBUACETAO2 + HCl | 2.07E-11 | NIST; Branch ratio refer to OH |
| OrCl.222 | SBUTACET + Cl --> SBUACETBO2 + HCl | 2.07E-11 | NIST; Branch ratio refer to OH |
| OrCl.223 | TBUACET + Cl --> MCOOTBO2 + HCl | 1.37E-11 | NIST; Branch ratio refer to OH |
| OrCl.224 | TBUACET + Cl --> TBOCOCH2O2 + HCl | 2.72E-12 | NIST; Branch ratio refer to OH |
| OrCl.225 | CH3OCH3 + Cl --> CH3OCH2O2 + HCl | 1.80E-10 | NIST |
| OrCl.226 | DIETETHER + Cl --> DIETETO2 + HCl | 2.38E-10 | NIST; Branch ratio refer to OH |
| OrCl.227 | DIETETHER + Cl --> ETOC2O2 + HCl | 1.85E-11 | NIST; Branch ratio refer to OH |
| OrCl.228 | MTBE + Cl --> MTBEAO2 + HCl | 7.14E-11 | NIST; Branch ratio refer to OH |
| OrCl.229 | MTBE + Cl --> MTBEBO2 + HCl | 6.86E-11 | NIST; Branch ratio refer to OH |
| OrCl.230 | DIIPRETHER + Cl --> DIIPRETO2 + HCl | 1.32E-10 | NIST; Branch ratio refer to OH |
| OrCl.231 | DIIPRETHER + Cl --> IPROMC2O2 + HCl | 2.77E-11 | NIST; Branch ratio refer to OH |
| OrCl.232 | ETBE + Cl --> ETBEAO2 + HCl | 2.70E-11 | NIST; Branch ratio refer to OH |
| OrCl.233 | ETBE + Cl --> ETBEBO2 + HCl | 1.14E-10 | NIST; Branch ratio refer to OH |
| OrCl.234 | ETBE + Cl --> ETBECO2 + HCl | 9.00E-12 | NIST; Branch ratio refer to OH |
| OrCl.235 | CH3Br + Cl --> CH2BrO2 + HCl | 4.28E-13 | NIST |
| OrCl.236 | APINENE + Cl --> OLEClO2 | 4.70E-10 | NIST |
| OrCl.237 | BPINENE + Cl --> OLEClO2 | 3.80E-10 | NIST |
| OrCl.238 | LIMONENE + Cl --> OLEClO2 | 6.40E-10 | NIST |
| OrCl.239 | DMM + Cl --> DMMAO2 + HCl | 9.86E-11 | NIST; Branch ratio refer to OH |
| OrCl.240 | DMM + Cl --> DMMBO2 + HCl | 3.65E-11 | NIST; Branch ratio refer to OH |
| OrCl.241 | DMC + Cl --> DMCO2 + HCl | 2.31E-12 | NIST |
| OrCl.242 | ETHOX + Cl --> ETHOXO2 | 2.82E-11 | NIST |
| **Br + VOCs** |  |  |  |
| **No.** | **Reaction** | **Rate constant** (cm^3^ molecule^-1^ s^-1^) at 298K, 1 atm | **Reference** |
| OrBr.1 | CHEX + Br --> CHEXO2 + HBr | 1.02E-17 | NIST |
| OrBr.2 | CH3OH + Br --> HBr + HCHO + HO2 | 1.68E-17 | NIST |
| OrBr.3 | C2H5OH + Br --> HBr + CH3CHO + HO2 | 9.22E-15 | NIST |
| OrBr.4 | NPROPOL + Br --> HBr + C2H5CHO + HO2 | 8.30E-15 | NIST |
| OrBr.5 | IPROPOL + Br --> CH3COCH3 + HBr + HO2 | 4.60E-14 | NIST |
| OrBr.6 | CH3OOH + Br --> HBr + CH3O2 | 1.18E-14 | NIST |
| OrBr.7 | C2H2 + Br --> CHOBr + CO + HO2 | 1.45E-15 | NIST |
| OrBr.8 | C2H4 + Br --> BrETAL | 1.60E-13 | NIST |
| OrBr.9 | C3H6 + Br --> C3H5O2 + HBr | 3.60E-12 | NIST |
| OrBr.10 | BUT1ENE + Br --> OLEBrO2 | 3.40E-12 | NIST |
| OrBr.11 | TBUT2ENE + Br --> OLEBrO2 | 6.46E-12 | NIST |
| OrBr.12 | CBUT2ENE + Br --> OLEBrO2 | 6.31E-12 | NIST |
| OrBr.13 | MEPROPENE + Br --> OLEBrO2 | 3.40E-12 | refer to kBr/kOH ratio of ME2BUT1ENE |
| OrBr.14 | PENT1ENE + Br --> OLEBrO2 | 1.52E-11 | refer to ME2BUT1ENE |
| OrBr.15 | CPENT2ENE + Br --> OLEBrO2 | 1.52E-11 | refer to ME2BUT0ENE |
| OrBr.16 | TPENT2ENE + Br --> OLEBrO2 | 1.52E-11 | refer to ME2BUT1ENE |
| OrBr.17 | ME2BUT1ENE + Br --> OLEBrO2 | 1.52E-11 | NIST |
| OrBr.18 | ME3BUT1ENE + Br --> OLEBrO2 | 1.52E-11 | refer to ME2BUT1ENE |
| OrBr.19 | ME2BUT2ENE + Br --> OLEBrO2 | 1.91E-11 | NIST |
| OrBr.20 | HEX1ENE + Br --> OLEBrO2 | 7.81E-13 | NIST |
| OrBr.21 | DM23BU2ENE + Br --> OLEBrO2 | 8.17E-11 | NIST |
| OrBr.22 | MVK + Br --> OLEBrO2 | 1.88E-11 | NIST |
| OrBr.23 | MACR + Br --> OLEBrO2 | 1.28E-11 | NIST |
| OrBr.24 | ACR + Br --> OLEBrO2 | 1.03E-12 | NIST |
| OrBr.25 | C4ALDB + Br --> OLEBrO2 | 6.80E-12 | Refer to kBr/kOH ratio of average of MVK, MACR and ACR |
| OrBr.26 | OLEBrO2 + HO2 = OLEBrO2H | 1.61E-11 | Refer to OLEClO2 |
| OrBr.27 | OLEBrO2 + NO --> OLEBrCHO + NO2 + HO2 | 9.04E-12 | Refer to OLEClO2 |
| OrBr.28 | OLEBrO2 + NO3 --> OLEBrCHO + NO2 + HO2 | 2.30E-12 | Refer to OLEClO2 |
| OrBr.29 | OLEBrO2 --> OLEBrCHO + HO2 | 7.22E-08 | Refer to OLEClO2 |
| OrBr.30 | OLEBrO2 = DUMMY | 4.81E-08 | Refer to OLEClO2 |
| OrBr.31 | OLEBrCHO + NO3 --> OLEBrCO3 + HNO3 | 1.50E-14 | Refer to OLEClO2 |
| OrBr.32 | OLEBrCHO + OH --> OLEBrCO3 | 2.85E-11 | Refer to OLEClO2 |
| OrBr.33 | OLEBrCHO --> OLEBrO2 + CO + HO2 | 2.00E-12*RO2 | Refer to OLEClO2 |
| OrBr.34 | OLEBrCO3 + HO2 --> DUMMY | 1.39E-11 | Refer to OLEClO2 |
| OrBr.35 | OLEBrCO3 + NO --> OLEBrO2 + NO2 | 1.98E-11 | Refer to OLEClO2 |
| OrBr.36 | OLEBrCO3 + NO3 --> OLEBrO2 + NO2 | 4.00E-12 | Refer to OLEClO2 |
| OrBr.37 | OLEBrCO3 --> OLEBrO2 | 4.21E-07 | Refer to OLEClO2 |
| OrBr.38 | OLEBrCO3 = DUMMY | 1.80E-07 | Refer to OLEClO2 |
| OrBr.39 | OLEBrCO3 + NO2 --> OLEBrPAN | 8.99E-12 | Refer to OLEClO2 |
| OrBr.40 | OLEBrPAN --> OLEBrCO3 + NO2 | 4.32E-04 | Refer to OLEClO2 |
| OrBr.41 | HCHO + Br --> HBr + CO + HO2 | 1.10E-12 | NASA |
| OrBr.42 | CH3CHO + Br --> HBr + CH3CO3 | 3.84E-12 | NASA |
| OrBr.43 | C2H5CHO + Br --> C2H5CO3 + HBr | 7.42E-12 | Ramacher et al. [36] |
| OrBr.44 | C3H7CHO + Br --> C3H7CO3 + HBr | 9.39E-12 | Ramacher et al. [36] |
| OrBr.45 | IPRCHO + Br --> IBUTALBO2 + HBr | 5.67E-13 | NIST; Branch ratio refer to OH |
| OrBr.46 | IPRCHO + Br --> IBUTALCO2 + HBr | 6.20E-13 | NIST; Branch ratio refer to OH |
| OrBr.47 | IPRCHO + Br --> IPRCO3 + HBr | 9.31E-12 | NIST; Branch ratio refer to OH |
| OrBr.48 | C4H9CHO + Br --> C4CHOBO2 + HBr | 2.17E-12 | Refer to kBr/kOH ratio of average of C2H5CHO, C3H7CHO and IPRCHO; Branch ratio refer to OH |
| OrBr.49 | C4H9CHO + Br --> C4H9CO3 + HBr | 9.24E-12 | Refer to kBr/kOH ratio of average of C2H5CHO, C3H7CHO and IPRCHO; Branch ratio refer to OH |
| OrBr.50 | BENZAL + Br --> C6H5CO3 + HBr | 5.02E-12 | Refer to kBr/kOH ratio of average of C2H5CHO, C3H7CHO and IPRCHO; |
| OrBr.51 | GLYOX + Br --> HCOCO + HBr | 3.88E-12 | Refer to kBr/kOH ratio of average of C2H5CHO, C3H7CHO and IPRCHO; |
| OrBr.52 | MGLYOX + Br --> HBr + CO + CH3CO3 | 5.23E-12 | Refer to kBr/kOH ratio of average of C2H5CHO, C3H7CHO and IPRCHO; |
| OrBr.53 | MACR + Br --> MACO3 + HBr | 1.05E-11 | NIST |
| OrBr.54 | ACR + Br --> ACRO2 + HBr | 2.18E-12 | NIST |
| OrBr.55 | C4ALDB + Br --> C3DBCO3 + HBr | 6.80E-12 | Refer to kBr/kOH ratio of average of MACR and ACR; |
| OrBr.56 | TOLUENE + Br --> C6H5CH2O2 + HBr | 1.30E-14 | NIST |
| OrBr.57 | OXYL + Br --> OXYLO2 + HBr | 8.90E-14 | NIST |
| OrBr.58 | MXYL + Br --> MXYLO2 + HBr | 6.59E-14 | NIST |
| OrBr.59 | PXYL + Br --> PXYLO2 + HBr | 9.00E-14 | NIST |
| OrBr.60 | TM123B + Br --> TM123BO2 + HBr | 4.80E-13 | NIST |
| OrBr.61 | TM124B + Br --> TM124BO2 + HBr | 4.80E-13 | refer to TM123B |
| OrBr.62 | TM135B + Br --> TMBO2 + HBr | 4.80E-13 | refer to TM123B |
| OrBr.63 | C4H6 + Br --> OLEBrO2 | 5.75E-11 | NIST |
| OrBr.64 | C5H8 + Br --> OLEBrO2 | 7.42E-11 | NIST |
| OrBr.65 | APINENE + Br --> OLEBrO2 | 2.23E-11 | NIST |
| OrBr.66 | BPINENE + Br --> OLEBrO2 | 2.86E-11 | NIST |

Supplementary Table 3: Chlorine and bromine related photochemical reactions in the box model. Jmax values for Dec 10 are shown as an example. The photolysis rate was calculated via the TUV model and scaled to measure J_NO2_.

| No | Reaction | Jmax on Dec 10 (s^-1^) | Lifetime |
| --- | --- | --- | --- |
| Photo.1 | Cl_2_ --> 2 Cl | 1.29E-03 | 12.9 min |
| Photo.2 | ClO --> O + Cl | 1.19E-05 | 23 hour |
| Photo.3 | OClO --> O + ClO | 4.81E-02 | 21 s |
| Photo.4 | HOCl --> Cl + OH | 1.52E-04 | 1.8 hour |
| Photo.5 | NOCl --> NO + Cl | 1.35E-03 | 12 min |
| Photo.6 | ClNO_2_ --> NO_2_ + Cl | 2.55E-04 | 1.1 hour |
| Photo.7 | ClONO_2_ --> Cl + NO_3_ | 2.20E-05 | 13 hour |
| Photo.8 | ClONO_2_ --> ClO + NO_2_ | 3.40E-06 | 3.4 days |
| Photo.9 | Cl_2_O_2_ --> 2 Cl | 9.23E-04 | 18 min |
| Photo.10 | Br_2_ --> 2 Br | 5.82E-03 | 2.9 min |
| Photo.11 | BrO --> O + Br | 2.04E-02 | 49 s |
| Photo.12 | HOBr --> Br + OH | 9.72E-04 | 17 min |
| Photo.13 | BrNO_2_ --> Br + NO_2_ | 2.40E-03 | 6.9 min |
| Photo.14 | BrNO_3_ --> NO_2_ + BrO | 9.94E-05 | 2.8 hour |
| Photo.15 | BrNO_3_ --> Br + NO_3_ | 5.63E-04 | 30 min |
| Photo.16 | BrCl --> Br + Cl | 4.38E-03 | 3.8 min |

Supplementary Table 4: Parameter inputs in the box model for the halogen impact evaluation. All listed parameters (except for CH_4_ and H_2_) in the table were the concurrent measurement data at our site. The calculation of the average value and standard deviation was based on the measurement period during 9-31 December 2017. Concentrations of CH_4_ and H_2_ were kept constant at values of 2000 ppbv [25] and 500 ppbv [26], respectively. The VOCs species name is presented in MCM style.

| No | Parameter | Time resolution | Average value ± Standard deviation |
| --- | --- | --- | --- |
| 1 | Temperature | 1 min | -0.1±2.6 ℃ |
| 2 | RH | 1 min | 43±5.2% |
| 3 | Surface Area Density | 1 min | 5034±750 μm^2^/cm^3^ |
| 4 | JNO2 | 1 min | 0.00096±0.0014 s^-1^ |
| 5 | NO | 1 min | 53±31 ppbv |
| 6 | NO2 | 1 min | 30.1±6.0 ppbv |
| 7 | O3 | 1 min | 8.5±5.3 ppbv |
| 8 | CO | 1 min | 2398±660 ppbv |
| 9 | SO2 | 1 min | 13.8±4.6 ppbv |
| 10 | NH3 | 1 min | 28.1±11.8 ppbv |
| 11 | N2O5 | 1 min | 0.01±0.01 ppbv |
| 12 | ClNO2 | 1 min | 0.07±0.02 ppbv |
| 13 | Cl2 | 1 min | 0.04±0.01 ppbv |
| 14 | HOBR | 1 min | 0.04±0.01 ppbv |
| 15 | BRCL | 1 min | 0.07±0.01 ppbv |
| 16 | BR2 | 1 min | 0.003±0.001 ppbv |
| 17 | HONO* | 1 min | 2.36±0.77 ppbv |
| 18 | H2O2* | 1 min | 0.18±0.13 ppbv |
| 19 | C2H6 | 1 hour | 14.3±4.9 ppbv |
| 20 | C2H4 | 1 hour | 1.7±0.7 ppbv |
| 21 | C3H8 | 1 hour | 5.0±1.4 ppbv |
| 22 | C3H6 | 1 hour | 4.4±2.0 ppbv |
| 23 | IC4H10 | 1 hour | 1.2±0.2 ppbv |
| 24 | NC4H10 | 1 hour | 2.6±0.6 ppbv |
| 25 | C2H2 | 1 hour | 5.3±1.7 ppbv |
| 26 | TBUT2ENE | 1 hour | 0.24±0.13 ppbv |
| 27 | BUT1ENE | 1 hour | 0.67±0.32 ppbv |
| 28 | CBUT2ENE | 1 hour | 0.11±0.02 ppbv |
| 29 | IC5H12 | 1 hour | 0.19±0.10 ppbv |
| 30 | NC5H12 | 1 hour | 1.02±0.24 ppbv |
| 31 | CH3CL | 1 hour | 0.44±0.06 ppbv |
| 32 | VINCL | 1 hour | 0.04±0.01 ppbv |
| 33 | C4H6 | 1 hour | 0.24±0.12 ppbv |
| 34 | CH3CHO | 1 hour | 2.14±0.36 ppbv |
| 35 | CH3BR | 1 hour | 0.008±0.001 ppbv |
| 36 | CH3CH2CL | 1 hour | 0.03±0.01 ppbv |
| 37 | PENT1ENE | 1 hour | 0.16±0.08 ppbv |
| 38 | TPENT2ENE | 1 hour | 0.11±0.07 ppbv |
| 39 | C5H8 | 1 hour | 0.11±0.06 ppbv |
| 40 | ACR | 1 hour | 0.24±0.08 ppbv |
| 41 | CPENT2ENE | 1 hour | 0.05±0.03 ppbv |
| 42 | C2H5CHO | 1 hour | 0.29±0.04 ppbv |
| 43 | CCL2CH2 | 1 hour | 0.002±0.001 ppbv |
| 44 | CH3COCH3 | 1 hour | 2.0±0.3 ppbv |
| 45 | CH2CL2 | 1 hour | 1.4±0.4 ppbv |
| 46 | M23C4 | 1 hour | 0.05±0.01 ppbv |
| 47 | M2PE | 1 hour | 0.32±0.08 ppbv |
| 48 | M3PE | 1 hour | 0.24±0.05 ppbv |
| 49 | MTBE | 1 hour | 0.10±0.02 ppbv |
| 50 | HEX1ENE | 1 hour | 0.20±0.11 ppbv |
| 51 | NC6H14 | 1 hour | 0.44±0.13 ppbv |
| 52 | MACR | 1 hour | 0.025±0.008 ppbv |
| 53 | CHCL2CH3 | 1 hour | 0.012±0.001 ppbv |
| 54 | C3H7CHO | 1 hour | 0.050±0.007 ppbv |
| 55 | MVK | 1 hour | 0.09±0.04 ppbv |
| 56 | MEK | 1 hour | 0.64±0.12 ppbv |
| 57 | CHCL3 | 1 hour | 0.52±0.11 ppbv |
| 58 | CH3CCL3 | 1 hour | 0.005±0.001 ppbv |
| 59 | M2HEX | 1 hour | 0.07±0.02 ppbv |
| 60 | CHEX | 1 hour | 0.11±0.02 ppbv |
| 61 | M3HEX | 1 hour | 0.08±0.01 ppbv |
| 62 | BENZENE | 1 hour | 3.56±1.22 ppbv |
| 63 | CH2CLCH2CL | 1 hour | 0.48±0.10 ppbv |
| 64 | NC7H16 | 1 hour | 0.20±0.07 ppbv |
| 65 | TRICLETH | 1 hour | 0.08±0.02 ppbv |
| 66 | MPRK | 1 hour | 0.06±0.02 ppbv |
| 67 | CL12PROP | 1 hour | 0.38±0.14 ppbv |
| 68 | C4H9CHO | 1 hour | 0.03±0.01 ppbv |
| 69 | DIEK | 1 hour | 0.03±0.01 ppbv |
| 70 | TOLUENE | 1 hour | 2.63±0.86 ppbv |
| 71 | NC8H18 | 1 hour | 0.14±0.05 ppbv |
| 72 | CH2CLCHCL2 | 1 hour | 0.03±0.01 ppbv |
| 73 | TCE | 1 hour | 0.09±0.02 ppbv |
| 74 | C5H11CHO | 1 hour | 0.30±0.03 ppbv |
| 75 | DIBRET | 1 hour | 0.001±0.001 ppbv |
| 76 | EBENZ | 1 hour | 0.35±0.12 ppbv |
| 77 | NC9H20 | 1 hour | 0.10±0.04 ppbv |
| 78 | MXYL | 1 hour | 0.83±0.30 ppbv |
| 79 | OXYL | 1 hour | 0.31±0.11 ppbv |
| 80 | STYRENE | 1 hour | 0.38±0.21 ppbv |
| 81 | IPBENZ | 1 hour | 0.02±0.01 ppbv |
| 82 | PBENZ | 1 hour | 0.03±0.01 ppbv |
| 83 | METHTOL | 1 hour | 0.12±0.05 ppbv |
| 84 | PETHTOL | 1 hour | 0.09±0.03 ppbv |
| 85 | NC10H22 | 1 hour | 0.08±0.03 ppbv |
| 86 | TM135B | 1 hour | 0.05±0.02 ppbv |
| 87 | OETHTOL | 1 hour | 0.06±0.02 ppbv |
| 88 | TM124B | 1 hour | 0.14±0.06 ppbv |
| 89 | DCBENE | 1 hour | 0.012±0.001 ppbv |
| 90 | TM123B | 1 hour | 0.008±0.003 ppbv |
| 91 | NC12H26 | 1 hour | 0.07±0.01 ppbv |
| 92 | CH3OH | 1 hour | 9.04±2.24 ppbv |
| 93 | CH4 | 1 hour | 2000±0 ppbv |
| 94 | HCHO | 2 hour | 3.55±1.08 ppbv |
| 95 | BENZAL | 2 hour | 0.18±0.03 ppbv |
| 96 | OXYLAL | 2 hour | 0.12±0.04 ppbv |
| 97 | MXYLAL | 2 hour | 1.35±0.80 ppbv |

*The HONO and H_2_O_2_ data obtained from the instrument was averaged to 10 minutes to avoid the diffusion effect in the analyzer for wet chemistry technique. The value input to the model was interpreted into 1-minute data.

**Supplementary Table 5: The simplified halogen heterogeneous reactions used for estimation of BrCl production**

| No. | Reaction | Uptake coefficient | Reference |
| --- | --- | --- | --- |
| Het.1 | BrONO2 + H2O --> HOBr + HNO3 | γ=0.4 | Hanson and Ravishankara [28] |
| Het.2 | HOBr + Cl- + H+--> BrCl + H2O | γ=0.05-0.2 | Hanson and Ravishankara [28] |
| Het.3 | ClONO2 + H2O --> HOCl + HNO3 | γ=0.0244 | Schmidt et al. [37] |
| Het.4 | HOCl + Br- --> BrCl + H2O | γ=0.01 | Huff and Abbatt [38] |

REFERENCES

1. Liao, J, Huey, LG, Liu, Z*, et al.* High levels of molecular chlorine in the Arctic atmosphere. *Nat. Geosci*. 2014; **7**: 91-4.

2. Le Breton, M, Bannan, TJ, Shallcross, DE*, et al.* Enhanced ozone loss by active inorganic bromine chemistry in the tropical troposphere. *Atmos. Environ.* 2017; **155**: 21-8.

3. Tham, YJ, Wang, Z, Li, Q*, et al.* Significant concentrations of nitryl chloride sustained in the morning: investigations of the causes and impacts on ozone production in a polluted region of northern China. *Atmos. Chem. Phys.* 2016; **16**: 14959-77.

4. Wang, T, Tham, YJ, Xue, L*, et al.* Observations of nitryl chloride and modeling its source and effect on ozone in the planetary boundary layer of southern China. *J. Geophys. Res. Atmos.* 2016; **121**: 2476-89.

5. Neuman, JA, Nowak, JB, Huey, LG*, et al.* Bromine measurements in ozone depleted air over the Arctic Ocean. *Atmos. Chem. Phys.* 2010; **10**: 6503-14.

6. Buys, Z, Brough, N, Huey, LG*, et al.* High temporal resolution Br_2_, BrCl and BrO observations in coastal Antarctica. *Atmos. Chem. Phys.* 2013; **13**: 1329-43.

7. Liu, X, Qu, H, Huey, LG*, et al.* High levels of daytime molecular chlorine and nitryl chloride at a rural site on the North China Plain. *Environ. Sci. Technol.* 2017; **51**: 9588-95.

8. Oldridge, NW, Abbatt, JPD. Formation of Gas-Phase Bromine from Interaction of Ozone with Frozen and Liquid NaCl/NaBr Solutions: Quantitative Separation of Surficial Chemistry from Bulk-Phase Reaction. *J. Phys. Chem. A.* 2011; **115**: 2590-8.

9. Artiglia, L, Edebeli, J, Orlando, F*, et al.* A surface-stabilized ozonide triggers bromide oxidation at the aqueous solution-vapour interface. *Nat. Commun.* 2017; **8**: 700.

10. Eger, PG, Helleis, F, Schuster, G*, et al.* Chemical ionization quadrupole mass spectrometer with an electrical discharge ion source for atmospheric trace gas measurement. *Atmos. Meas. Tech.* 2019; **12**: 1935-54.

11. Lee, BH, Lopez-Hilfiker, FD, Veres, PR*, et al.* Flight Deployment of a High-Resolution Time-of-Flight Chemical Ionization Mass Spectrometer: Observations of Reactive Halogen and Nitrogen Oxide Species. *J. Geophys. Res. Atmos.* 2018; **123**: 7670-86.

12. Liu, P, Zhang, C, Mu, Y*, et al.* The possible contribution of the periodic emissions from farmers' activities in the North China Plain to atmospheric water-soluble ions in Beijing. *Atmos. Chem. Phys.* 2016; **16**: 10097-109.

13. Li, H, Zhang, Q, Chen, C*, et al.* Wintertime aerosol chemistry and haze evolution in an extremely polluted city of the North China Plain: significant contribution from coal and biomass combustion. *Atmos. Chem. Phys.* 2017; **17**: 4751-68.

14. Liu, P, Zhang, C, Xue, C*, et al.* The contribution of residential coal combustion to atmospheric PM2. 5 in northern China during winter. *Atmos. Chem. Phys.* 2017; **17**: 11503-20.

15. Liu, HJ, Zhao, CS, Nekat, B*, et al.* Aerosol hygroscopicity derived from size-segregated chemical composition and its parameterization in the North China Plain. *Atmos. Chem. Phys.* 2014; **14**: 2525-39.

16. Ren, D, Zhao, F, Wang, Y*, et al.* Distributions of minor and trace elements in Chinese coals. *Int. J. Coal. Geol.* 1999; **40**: 109-18.

17. Peng, B-x. Study on Environmental Geochemistry of Bromine in Chinese Coals. *Doctoral Thesis*. Nanchang University; 2011.

18. Tang, Y, He, X, Cheng, A*, et al.* Occurrence and sedimentary control of sulfur in coals of China. *Journal of China Coal Society*. 2015; **40**: 1976-87.

19. Du, Q, Zhang, C, Mu, Y*, et al.* An important missing source of atmospheric carbonyl sulfide: Domestic coal combustion. *Geophys. Res. Lett.* 2016; **43**: 8720-7.

20. Natural Resources Defense Council. *China Dispersed Coal Governance Report 2017 (in Chinese)*. 2017, http://coalcap.nrdc.cn/pdfviewer/web/?15180772751437518672.pdf.

21. Sandu, A, Sander, R. Technical note: Simulating chemical systems in Fortran90 and Matlab with the Kinetic PreProcessor KPP-2.1. *Atmos. Chem. Phys.* 2006; **6**: 187-95.

22. Burkholder, J, Sander, S, Abbatt, J*, et al.* *Chemical kinetics and photochemical data for use in atmospheric studies: evaluation number 18*. Pasadena, CA: Jet Propulsion Laboratory, National Aeronautics and Space 2015.

23. Xue, LK, Saunders, SM, Wang, T*, et al.* Development of a chlorine chemistry module for the Master Chemical Mechanism. *Geosco. Model. Dev.* 2015; **8**: 3151-62.

24. Saunders, SM, Jenkin, ME, Derwent, RG*, et al.* Protocol for the development of the Master Chemical Mechanism, MCM v3 (Part A): tropospheric degradation of non-aromatic volatile organic compounds. *Atmos. Chem. Phys.* 2003; **3**: 161-80.

25. Tan, Z, Fuchs, H, Lu, K*, et al.* Radical chemistry at a rural site (Wangdu) in the North China Plain: observation and model calculations of OH, HO_2_ and RO_2_ radicals. *Atmos. Chem. Phys.* 2017; **17**: 663-90.

26. Ehhalt, DH, Rohrer, F. The tropospheric cycle of H_2_: a critical review. *Tellus B: Chemical and Physical Meteorology*. 2009; **61**: 500-35.

27. Zhu, X, Tang, G, Guo, J*, et al.* Mixing layer height on the North China Plain and meteorological evidence of serious air pollution in southern Hebei. *Atmos. Chem. Phys.* 2018; **18**: 4897-910.

28. Hanson, DR, Ravishankara, AR. Heterogeneous chemistry of bromine species in sulfuric acid under stratospheric conditions. *Geophys. Res. Lett.* 1995; **22**: 385-8.

29. Ammann, M, Cox, RA, Crowley, JN*, et al.* Evaluated kinetic and photochemical data for atmospheric chemistry: Volume VI – heterogeneous reactions with liquid substrates. *Atmos. Chem. Phys.* 2013; **13**: 8045-228.

30. Hissler, C, Stille, P, Krein, A*, et al.* Identifying the origins of local atmospheric deposition in the steel industry basin of Luxembourg using the chemical and isotopic composition of the lichen Xanthoria parietina. *Sci. Total. Environ.* 2008; **405**: 338-44.

31. Wang, Z, Wang, W, Tham, YJ*, et al.* Fast heterogeneous N2O5 uptake and ClNO2 production in power plant and industrial plumes observed in the nocturnal residual layer over the North China Plain. *Atmos. Chem. Phys.* 2017; **17**: 12361-78.

32. International Energy Agency. *International Energy Agency World Energy Statistics, 1960-2018*. 2019, http://doi.org/10.5257/iea/wes/2019.

33. Nicovich, JM, Wine, PH. Kinetics of the reactions of O(3P) and Cl(2P) with HBr and Br2. *Int. J. Chem. Kinet.* 1990; **22**: 379-97.

34. Michalowski, BA, Francisco, JS, Li, S-M*, et al.* A computer model study of multiphase chemistry in the Arctic boundary layer during polar sunrise. *J. Geophys. Res. Atmos.* 2000; **105**: 15131-45.

35. Sander, R, Baumgaertner, A, Gromov, S*, et al.* The atmospheric chemistry box model CAABA/MECCA-3.0. *Geosco. Model. Dev.* 2011; **4**: 373-80.

36. Ramacher, B, Orlando, JJ, Tyndall, GS. Temperature-dependent rate coefficient measurements for the reaction of bromine atoms with a series of aldehydes. *Int. J. Chem. Kinet.* 2000; **32**: 460-5.

37. Schmidt, JA, Jacob, DJ, Horowitz, HM*, et al.* Modeling the observed tropospheric BrO background: Importance of multiphase chemistry and implications for ozone, OH, and mercury. *J. Geophys. Res. Atmos.* 2016; **121**: 11819-35.

38. Huff, AK, Abbatt, JPD. Gas-Phase Br_2_ Production in Heterogeneous Reactions of Cl_2_, HOCl, and BrCl with Halide−Ice Surfaces. *J. Phys. Chem. A.* 2000; **104**: 7284-93.
